# Hyaluronic acid synthesis is required for zebrafish tail fin regeneration

**DOI:** 10.1371/journal.pone.0171898

**Published:** 2017-02-16

**Authors:** Xiaohu Ouyang, Nicholas J. Panetta, Maya D. Talbott, Alexander Y. Payumo, Caroline Halluin, Michael T. Longaker, James K. Chen

**Affiliations:** 1 Department of Chemical and Systems Biology, Stanford University School of Medicine, Stanford, California, United States of America; 2 Department of Surgery, Stanford University School of Medicine, Stanford, California, United States of America; 3 Institute of Stem Cell Biology and Regenerative Medicine, Stanford University School of Medicine, Stanford, California, United States of America; 4 Department of Developmental Biology, Stanford University School of Medicine, Stanford, California, United States of America; University Zürich, SWITZERLAND

## Abstract

Using genome-wide transcriptional profiling and whole-mount expression analyses of zebrafish larvae, we have identified *hyaluronan synthase 3* (*has3*) as an upregulated gene during caudal fin regeneration. *has3* expression is induced in the wound epithelium within hours after tail amputation, and its onset and maintenance requires fibroblast growth factor, phosphoinositide 3-kinase, and transforming growth factor-ß signaling. Inhibition of hyaluronic acid (HA) synthesis by the small molecule 4-methylumbelliferone (4-MU) impairs tail regeneration in zebrafish larvae by preventing injury-induced cell proliferation. In addition, 4-MU reduces the expression of genes associated with wound epithelium and blastema function. Treatment with glycogen synthase kinase 3 inhibitors rescues 4-MU-induced defects in cell proliferation and tail regeneration, while restoring a subset of wound epithelium and blastema markers. Our findings demonstrate a role for HA biosynthesis in zebrafish tail regeneration and delineate its epistatic relationships with other regenerative processes.

## Introduction

Regenerative medicine has the potential to provide therapies that enable the repair or replacement of damaged tissues and organs. While mammals have limited regenerative capacity, other organisms can replace complex structures upon injury or amputation [1]. Understanding how these non-mammalian systems maintain this remarkable capability for self-renewal could provide insights into why these processes are more restricted in “higher” organisms and ultimately lead to strategies for their therapeutic reactivation.

The zebrafish has emerged as a powerful model for studying regeneration of the heart, spinal cord, liver, pancreas, retina, tail, and other tissues [2,3]. Among these regenerative structures, the adult zebrafish tail is unparalleled for its accessibility to amputation and relatively simple cellular organization [4–6]. The tail regeneration process involves several discrete steps: (1) epidermal cell migration to form a wound epithelium and subsequent signaling center called the apical epidermal cap; (2) cell dedifferentiation to form a multipotent mesenchymal structure called the blastema; (3) proliferation of the proximal blastema; and (4) differentiation of these cells to replace the amputated tissues. Several signaling molecules, including fibroblast growth factors (FGFs) [7,8], bone morphogenetic proteins (BMPs) [9,10], Hedgehog (Hh) ligands [9], Wnts [11,12], insulin-like growth factors (IGFs) [13], activinßA [14] and retinoic acids (RAs) [15] are involved in one or more of the regenerative steps [5,16]. In addition to targeted studies of known signaling molecules, systematic methods such temperature-sensitive mutant screening [8], suppression subtractive hybridization [17], differential display RT-PCR [17], and microarray-based transcriptional profiling [18] have identified new genetic regulators expressed in the wound epithelium and blastema.

Zebrafish larvae can also regenerate their caudal fins, in a process similar to that of the adult tail [19,20]. Larval tail regeneration has been an attractive alternate model, since this life stage enables studies with larger sample sizes and shorter experimental timeframes (caudal fin regrowth is morphologically complete three days after amputation). Moreover, zebrafish larvae are amenable to transient genetic manipulations, chemical treatments, and live imaging. For example, using small-molecule compound libraries, it was demonstrated that receptors for aryl hydrocarbons [21], glucocorticoids [22] and ErbB2/3 ligands [23] are required for tail regeneration. Transcriptional profiling has also been used to discover genes that are differentially expressed in response to larval tail amputation, many of which have been found to regulate caudal fin regrowth [24,25]. For example, *aldehyde dehydrogenase 1a2* (*aldh1a2*) and *jun B proto-oncogene b* (*junbb*) are upregulated in tail-amputated larvae, and knockdown of these genes by morpholino antisense oligonucleotides inhibits caudal fin regeneration [24–26].

To identify and study novel regulators of larval tail regrowth, we have conducted a genome-wide, microarray-based survey of the regenerative transcriptome, followed by whole-mount *in situ* hybridization analysis of selected upregulated transcripts. Through this approach, we have identified a number of genes that are specifically expressed in distinct domains of the regenerating tail. Among these genetic regulators is *hyaluronan synthase 3* (*has3*), one of three biosynthetic enzymes in vertebrates that catalyze the synthesis of hyaluronic acid (HA). Although this glycosaminoglycan was first appreciated for its unique hydrodynamic properties, more recent studies have revealed important roles for these glucuronic acid/N-acetylglucosamine disaccharide polymers in embryonic development, wound healing, tissue repair, and tumor development [27–29].

We demonstrate here that *has3* expression is induced within 6 hours after tail amputation at 2 days post fertilization (dpf), reaching maximum levels within 1 day and then declining thereafter. The onset and maintenance of *has3* transcription is restricted to the wound epithelium and requires several signaling pathways, including those initiated by FGFs, phosphoinositide 3-kinase (PI3K), and transforming growth factor-ß (TGFß). Moreover, chemical inhibition of HA synthesis within the first 24 hours after amputation specifically suppresses regenerative cell proliferation and tail regrowth, as well as expression of the wound epithelium marker *distal-less homeobox gene 5a* (*dlx5a*) and blastemal genes previously associated with tail regeneration, *aldh1a2* and *junbb*. We also find that glycogen synthase 3-kinase (GSK3) inhibition rescues 4-MU-mediated defects in cell proliferation and tail regeneration; however, only *dlx5a* and *junbb* expression are restored. Taken together, our results reveal an essential role for HA in zebrafish tail regrowth that may reflect an evolutionary conserved mechanism of tissue regeneration.

## Materials and methods

### Zebrafish husbandry and larval tail amputation

Wild type AB and *Tg*(*TOP*:*GFP*) zebrafish were obtained from the Zebrafish International Resource Center and maintained, mated, and staged according to standard protocols [30,31]. For amputation experiments, zebrafish larvae were placed on a 10-cm plate filled with 1.5% agarose, and the tails were amputated at a position immediately caudal to the notochord using a needle blade (Fine Science Tools, Item Number 10318–14). Amputated larvae were then raised in E3 media in at 28°C. Zebrafish adult tail fin amputations were performed as previously described [11], after which fish were returned to 28°C aquarium water. Both larval and adult fish fins were subsequently fixed with 4% paraformaldehyde in PBS at the appropriate time points for further analyses. All zebrafish experiments were approved by the Institutional Animal Care and Use Committee at Stanford University.

### Expression profiling and data analysis

For profiling the transcriptional changes associated with tail regeneration, posterior tissues of tail-amputated larvae and uncut siblings were collected on ice at 1 day post amputation (dpa; 3 dpf). Total RNA was extracted from these tissues using an RNeasy Mini Kit (Invitrogen), and genomic DNA was removed using a DNA-free Kit (Applied Biosystems). Approximately 1 μg of total RNA was recovered from 150 tails for each condition, which was then amplified with a MessageAmp II aRNA Kit (Ambion). Typically at least 100 μg total RNA was recovered from two rounds of amplifications. The quantity and quality of total RNA were then assessed by 260-nm and 280-nm absorbance levels, and RNA integrity was evaluated with an Agilent 2100 Bioanalyzer.

cDNA synthesis and labeling, hybridization to the NimbleGen Zebrafish Gene Expression 385K array and microarray scanning were performed at the Stanford Functional Genomics Center, according to guidelines established by Roche NimbleGen. Data compilation was performed using NimbleGen software, and robust multiarray average (RMA) background correction and quartile normalization of raw.pair files were conducted with DNASTAR ArrayStar software. Probe signal intensities for cut and uncut samples were compared, and differentially expressed transcripts were identified using fold-change ≥ 1.5 and false discovery rate ≤ 0.1 as thresholds (moderated t-test, Benjamini-Hochberg correction). Microarray data for this study have been deposited in the Gene Expression Omnibus (GEO) database (NCBI) under accession number GSE72422.

### Whole-mount *In Situ* hybridization analysis

cDNAs encoding gene-specific sequences flanked with a T7 promoter were amplified using the PCR primers shown in S1 Table. Digoxigenin-labeled RNA probes (approximately 500–1100 bases long) were then *in vitro* transcribed from these PCR products using a MEGAscript T7 Kit (Invitrogen). Whole-mount *in situ* hybridizations were performed according to standard protocols [32].

### Pharmacological modulation of regenerative pathways

4-methylumbelliferone (4-MU), (2’Z,3’E)-6-Bromoindirubin-3′-oxime (BIO), SB431542, SP600125, dorsomorphin, and lithium chloride (LiCl) were purchased from Sigma-Aldrich; SB216763 from Santa Cruz Biotechnology; LY294002 from Cell Signaling Technologies; PD173074 from Selleck Chemicals; DAPT from Calbiochem. Cyclopamine was a gift from Infinity Pharmaceuticals. All small molecules were dissolved in dimethyl sulfoxide (DMSO) to prepare 1–200 mM stock solutions, which were stored at –20°C if not used immediately. For larval tail regeneration studies, small-molecule stock solutions were diluted with E3 embryo medium to achieve the appropriate working concentrations, and zebrafish larvae were added to this medium for the specified time period. The larvae were then transferred to fresh E3 media containing the compounds or an equivalent amount of DMSO vehicle on a daily basis, until they were collected and fixed at the appropriate time point for further analysis.

### *In vitro* studies of zebrafish *has3* and 4-MU activities

Zebrafish *has3* cDNA was PCR-amplified from a larval cDNA library and cloned into a pCMV6 expression vector. A pCMV6 vector containing human *HAS2* cDNA was purchased from OriGene. Both expression constructs were transfected into HEK293 cells in a 6-well plate, and the cells were cultured further for 24 hours with DMSO or increasing concentrations of 4-MU. HA secreted into the media was then analyzed by gel electrophoresis using modified published procedures [33,34]. Briefly, HA was purified by treating 2 mL of media with 500 μg of proteinase K (Roche) at 50°C for 60 min and precipitating the samples with 4 mL of ethanol at room temperature for 1 hour. After centrifugation at 18,000 *g* and 4°C for 30 min, the resulting pellets was resuspended in 40 μL of water. One sample derived from the DMSO-treated group was treated with 1 U/mL of hyaluronidase from *Streptomyces hyalurolyticus* (Sigma-Aldrich) to confirm its HA content, and all samples were then heated at 100°C for 20 min. After cooling to room temperature and brief pelleting insoluble debris by centrifugation, the supernatant (30 μL) was mixed with 4 μL of 2 M sucrose and 1 μL of 0.03% (w/v) bromophenol blue in TAE buffer, and 30 μL of the resulting sample was loaded into individual wells of a 0.5% agarose gel. The gel was electrophoresed at 50 V for 65 min in TAE buffer using the Mupid-exU system (Clontech), rinsed with water, and placed in 200 mL of 0.005% (v/v) Stains-All in 50% ethanol overnight in the dark. After rinsing with water and de-staining with an aqueous solution of 10% (v/v) ethanol, the gel was imaged with GE Typhoon 9410 imager using 633-nm excitation.

### Morpholino studies

To study Has3 function in zebrafish embryos, morpholino oligonucleotides targeting either the *has3* start codon (ATG-MO: 5’-CCGCAGTGCCAAAGCGAGAGGGCAT-3’) or the intron 2-exon 3 splice junction (i2e3-MO: 5’-ATCTGAAGGAAACAATGAACAGAGA-3’) were purchased from Gene Tools LLC. Morpholino solutions containing 0.1% (w/v) phenol red were microinjected into zebrafish zygotes (2 nL/embryo), and the embryos were cultured in E3 medium at 28°C. RNA missplicing in the *has3* i2e3-MO-injected embryos by was confirmed by RT-PCR using the following primers: 5’-CCTGATGTGGGAGGAGTTGGAGGA-3’ (forward) and 5’-GGACGCGGTTGGTGAGATGTCG-3’ (reverse).

### Cell proliferation analyses

Fixed zebrafish larvae were permeabilized in pre-cooled acetone at –20°C (7 min), washed with water (5 min), and PBX (PBS containing 0.1% Triton X-100, 2 x 5 min). The larvae were incubated in blocking solution (PBX containing 10% sheep serum and 0.5% BSA) for 30 min at room temperature and then incubated overnight at 4°C with Alexa Fluor 488-conjugated anti-phosphohistone H3 rabbit monoclonal antibody (1:750 dilution; Cell Signaling Technology, catalog number 3465). The larvae were then washed with PBX (6 x 15 min) and mounted for imaging.

### Imaging of zebrafish embryos and larvae

Live embryos: chorions were manually removed from 28 hpf-embryos, which were immobilized in E3 medium containing 0.7% (w/v) low-melt agarose and 0.05% (w/v) tricaine. Brightfield images were acquired using a Leica M205FA fluorescence stereoscope equipped with a Leica DFC500 digital camera.

Fixed larvae: after whole-mount *in situ* hybridization or immunostaining, regenerating larval tails were surgically removed and mounted on glass slides with cover glasses. Samples fixed in 4% paraformaldehyde or processed for *in situ* hybridization were mounted in 1X PBS containing 2% (w/v) methylcellulose, and samples processed for immunostaining staining were mounted in 80% glycerol containing 2.5% DABCO (Aldrich). *In situ* hybridization images were obtained with a Leica DM4500B epifluorescence microscope equipped with a 20x/0.5 NA objective and a QImaging Retiga-SRV digital camera. For samples stained with phosphohistone H3 antibody, both brightfield and fluorescence images were acquired using a Leica M205FA fluorescence stereoscope equipped with a Leica DFC500 digital camera. To quantify the regeneration phenotype in zebrafish larvae, we measured the fin areas posterior to the amputation line at 3 dpa (5 dpf) and then normalized them to the 5-dpf uncut control group to obtain tail regeneration percentage score. To quantify the effect of inhibitors on the expression of wound epidermis and blastema markers in the tail, we categorized each sample as “strong”, “weak” or “none” phenotypes based on the riboprobe staining intensities and gene expression patterns. The percent distribution of these phenotypes was then calculated for each compound treatment.

## Results

### Hyaluronic acid synthesis is upregulated during zebrafish tail regeneration

To identify novel molecular regulators of caudal fin regeneration, we compared the transcriptomes of posterior tissues isolated from tail-amputated and uncut zebrafish larvae. Caudal fins were amputated at 2 dpf, and total RNA was isolated at 1 dpa for analysis using a NimbleGen Zebrafish Gene Expression 385K Array (~37,000 zebrafish transcripts). Through this approach, we identified 97 upregulated and 45 downregulated genes in the regenerating tissue (fold change ≥ 1.5; false discovery rate ≤ 0.1) (S2 Table).

We next selected a subset of upregulated genes with catalytic and/or signaling functions and examined their expression patterns during tail regeneration. As assessed by whole-mount *in situ* hybridization, a number of these genes were regiospecifically transcribed in amputated tails, with little or no detectable expression in uncut caudal fins (S1 and S2 Figs). For example, Bcl-2 family member *bcl2l10*, fibroblast growth factor *fgf20a*, Jun transcription factor *junba*, and matrix metalloproteinase *mmp9* were expressed in partially overlapping distal regions that likely represent wound epithelium. Aldehyde dehydrogenase *aldh1a2*, sorting nexin *snx18a*, suppressor of cytokine signaling *socs3b*, and extracellular matrix proteoglycan *vcana* were expressed in more proximal domains that are indicative of blastema-like mesenchymal cells.

Among the genes upregulated during caudal fin regeneration was *hyaluronan synthase 3* (*has3*) (S1 Fig), a multipass transmembrane enzyme that generates HA from uridine diphosphate glucose (UDP)-activated glucuronic acid (GlcUA) and N-acetylglucosamine (GlcNAc). In vertebrates, HA is synthesized by three structurally homologous enzymes (HAS1, HAS2, and HAS3), which produce polysaccharides of different sizes and at different rates [35]. Newly synthesized long HA (n-HA) can then be degraded by hyaluronidases (e.g., HYAL1-4, PH20, HYALP1) to generate smaller polymers (o-HA) with distinct biological activities [36,37]. HA has been shown to influence cell behavior during embryonic development and tumor development by interacting with cell-surface receptors such as the type I transmembrane protein CD44 and hyaluronan-mediated motility receptor (HMMR) [27]. HA may also be a key factor in scarless fetal wound healing, as this glycosaminoglycan is highly upregulated for weeks after fetal injury (versus days in adults) [38], and fibrotic healing correlates with hyaluronidase activity [39]. More recently, HA has been implicated in tail regeneration in *Xenopus* tadpoles, as both *Has2* and *Hyal2* are transiently upregulated in the regenerative bud after tail amputation [40].

**Fig 1:**
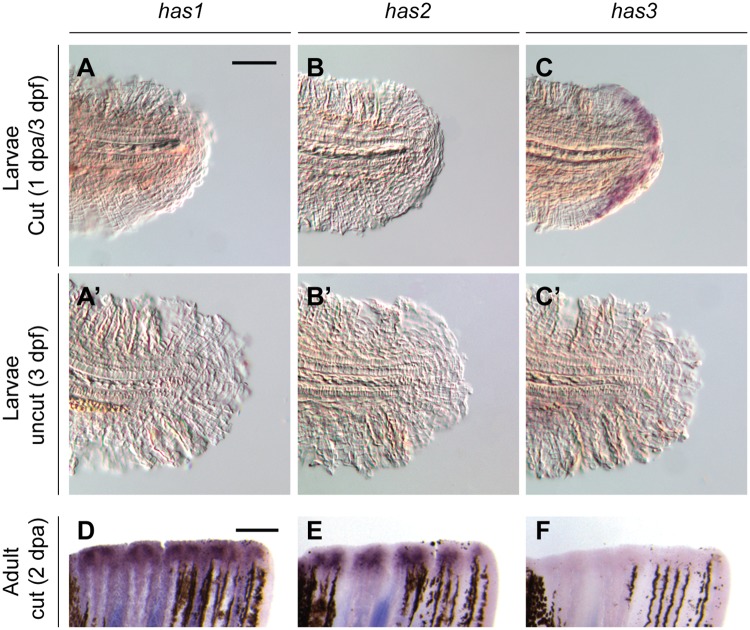
Zebrafish hyaluronan synthases are differentially expressed during larval and adult tail regeneration. Expression patterns of *has1*, *has2*, and *has3* in regenerating larval (**A-C**; 1 dpa) adult (**D-E**; 2 dpa) tails, as determined by whole-mount *in situ* hybridization. (**A’-C’**) Equivalently stained uncut controls. At least 30 larvae or 10 adult zebrafish were analyzed for each experimental condition, and phenotypic descriptions were based on a penetrance of > 80%. Scale bars: **A-C** and **A’-C’**, 100 μm; **D-E**: 300 μm.

Has2 is the only hyaluronan synthase expressed during the first day of zebrafish development, and its function is required for lateral cell migration during dorsal convergence [41]. Transcription of *has1* and *has3* is initiated by 2 dpf, although their physiological functions remain unknown [41]. To confirm that *has3* is the primary hyaluronan synthase expressed during larval tail regeneration, we examined the expression of each family member in tail-amputated larvae (3 dpf/1 dpa). In accordance with our microarray results, we did not observe *has1* or *has2* transcripts in the regenerative bud by whole-mount *in situ* hybridization (Fig 1A–1C). Only *has3* was expressed in the amputated tail, with transcripts localized to the wound epithelium. To determine whether one or more hyaluronan synthases are also upregulated during adult caudal fin regrowth, we conducted analogous whole-mount analyses of adult zebrafish at 2 dpa, by which time wound epidermis and blastema formation are complete [20]. In comparison to our observations in zebrafish larvae, we detected transcripts for *has1* and *has2*, but not *has3*, in the regenerating adult fin (Fig 1D and 1E). These results suggest that HA is also required for adult fin regeneration but synthesized through different enzyme isoforms.

**Fig 2:**
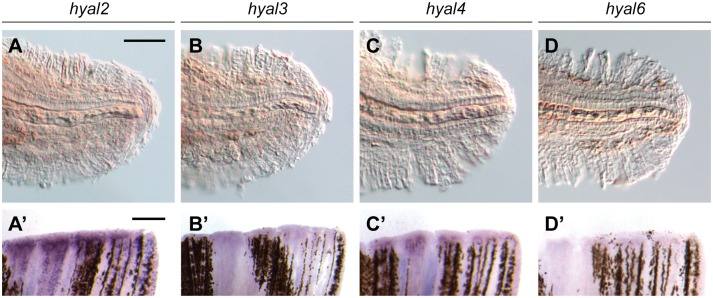
Zebrafish hyaluronidases are differentially expressed during larval and adult tail regeneration. Expression patterns of *hyal2*, *hyal3*, *hyal4*, and *hyal6* in regenerating larvae (**A-D**; 1 dpa) and adult (**A’-D’**; 2 dpa) tails. At least 30 larvae or 10 adult zebrafish were analyzed for each experimental condition, and phenotypic descriptions were based on a penetrance of > 80%. Scale bars: **A-D**, 100 μm; **A’-D’**: 300 μm.

Studies of vertebrate hyaluronan synthases have demonstrated that HAS3 generates shorter HA polysaccharides (0.1–1 MDa) than those synthesized by HAS1 (0.2–2 MDa) or HAS2 (> 2 MDa) [33,35,42], and biological activities of HA vary with size [36,37]. Since HA structure can be further regulated by endoglycosidic activity, we investigated whether amputated larval and adult tails also differentially express hyaluronidase family members, represented by zebrafish *hyal2*, *hyal3*, *hyal4*, and *hyal6*. While we could not detect transcripts for these hydrolytic enzymes in amputated larval caudal fins (Fig 2A–2D), we observed significant *hyal2* and *hyal4* expression in regenerating adult tails (2 dpa) (Fig 2A’–2D’). Taken together, these results raise the possibility that shorter forms of HA contribute to the regenerative process in both larval and adult zebrafish. In this model, Has3 would be the primary source of this HA subtype at larvae stages, whereas producing analogous oligosaccharides in adult fish would require the collective actions of Has1, Has2, Hyal2, Hyal4, and perhaps other hyaluronidases.

### Timing and regulation of *has3* expression during larval tail regeneration

**Fig 3:**
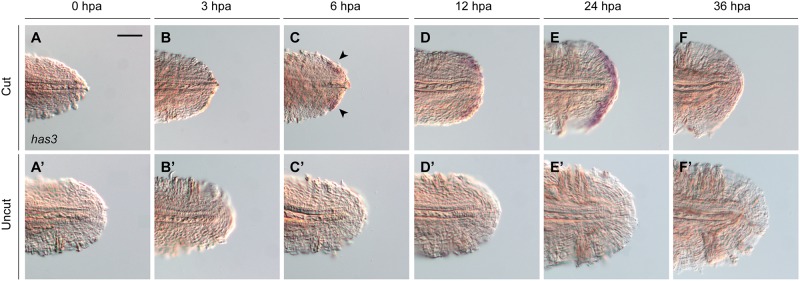
Dynamics of *has3* expression during larval tail regeneration. (**A-F**) Expression patterns of *has3* at different time points after caudal fin amputation at 2 dpf. Arrows mark the initial appearance of *has3* transcripts at 6 hpa, localized to dorsal and ventral sides regions of the regenerative bud. (**A’-F’**) Equivalently stained uncut controls at the same time points. At least 30 larvae were analyzed for each experimental condition, and phenotypic descriptions were based on a penetrance of > 80%. Scale bar: 100 μm.

Due to the experimental tractability of early-stage zebrafish, we focused on the roles of *has3* and HA in larval tail regeneration. We first determined the timing of *has3* expression in response to caudal fin amputation. Larval tails were cut at 2 dpf, and the regenerating tissues were fixed at different time points for analysis by whole-mount *in situ* hybridization. *has3* mRNA was first detected in dorsal and ventral regions of the wound epithelium at 6 hours post amputation (hpa), becoming more broadly expressed within the epidermis by 24 hpa (Fig 3). Expression of *has3* then declined and was largely extinguished by 36 hpa. Thus, *has3* activity is primarily upregulated during the first 24 hours of larval tail regeneration, during which the wound epithelium and blastema are formed [19].

**Fig 4:**
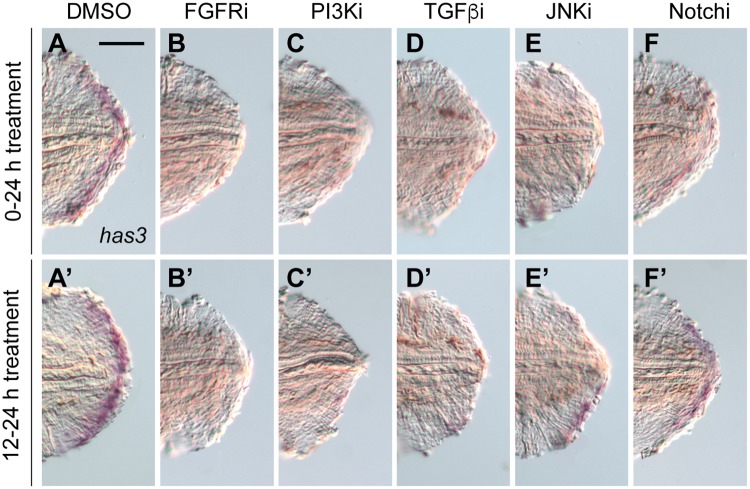
Multiple signaling pathways regulate the onset and maintenance of *has3* expression during larval tail regeneration. Expression of *has3* in 1-dpa (3-dpf) larval tails treated with the following signaling pathway inhibitors for the first 24 hours after amputation: (**A, A’**) 0.5% DMSO. (**B, B’**) 75 μM PD173074 (FGF). (**C, C’**) 10 μM LY294002 (PI3K). (**D, D’**) 50 μM SB431542 (TGFß). (**E, E’**) 5 μM SP600125 (JNK). (**F, F’**) 50 μM DAPT (Notch). At least 30 larvae were analyzed for each experimental condition, and phenotypic descriptions were based on a penetrance of > 80%. Scale bar: 100 μm.

We next investigated the molecular mechanisms that control *has3* transcription in the regenerative bud. Several signaling pathways have been shown to play critical roles in adult tail regrowth, including FGF, PI3K, TGFß, c-Jun N-terminal kinase (JNK), Notch, Hh, and BMP signaling [7–10,14,26,43–45]. We therefore examined the requirement for each pathway in larval tail regeneration, taking advantage of pathway-specific chemical inhibitors and the amenability of zebrafish larvae to pharmacological perturbations [9,14,26,43,45–47]. With the exception of the Hh signaling inhibitor cyclopamine and BMP receptor antagonist dorsomorphin, all other pathway blockers suppressed caudal fin regrowth (S3 Fig), underscoring the mechanistic similarities between larval and adult tail regeneration. Each of these active compounds also suppressed *has3* transcription when applied to zebrafish larvae for the first 24 hours after tail amputation (Fig 4A–4F). These results indicate that the onset and/or maintenance of *has3* expression requires multiple signaling pathways.

To better understand the timing by which these signaling mechanisms regulate *has3* expression, we also treated tail-amputated larvae with the pathway inhibitors from 12 to 24 hpa, several hours after *has3* transcription is initiated. We observed that inhibition of FGF, PI3K, or TGFß signaling was still able to abrogate *has3* expression, indicating that these pathways have specific roles in the maintenance of *has3* transcription (Fig 4A’–4D’). In comparison, *has3* expression could still be observed in tail-amputated larvae treated with JNK or Notch signaling antagonists during this time frame (Fig 4E’ and 4F’).

### Pharmacological inhibition of hyaluronic acid synthesis blocks larval tail regeneration

**Fig 5:**
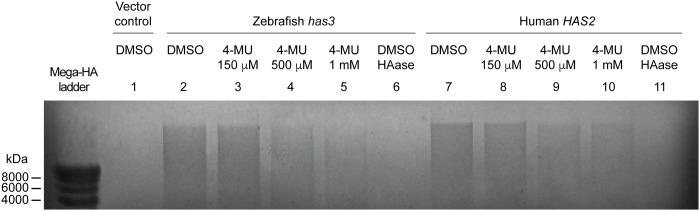
4-MU inhibits hyaluronan synthase-dependent HA production. Agarose gel electrophoresis and cationic dye staining of HA produced by HEK293 cells transfected with either zebrafish *has3* (lanes 2–5) or human *HAS2* (lanes 7–10) and then treated with varying doses of 4-MU. The structural identity of the stained HA was confirmed by *Streptomyces hyalurolyticus* hyaluronidase treatment (lanes 6 and 11).

Having established that *has3* upregulation is an early and highly regulated event during larval tail regeneration, we sought to determine its role in this process. It has been shown that cultured cells transfected with mammalian HAS genes are able to produce and secrete HA [33,35,48,49], and we first used this approach to confirm that zebrafish Has3 can similarly mediate HA synthesis. HEK293 cells transiently transfected with either zebrafish *has3* cDNA or a human *HAS2* cDNA control secreted HA into the culture media that could be detected with a cationic dye after agarose gel electrophoresis (Fig 5; lanes 2 and 7). Moreover, the oligosaccharide generated by these cells could be digested by *Streptomyces hyalurolyticus* hyaluronidase (Fig 5; lanes 6 and 11).

To investigate the role of Has3 in larval tail regeneration, we first obtained morpholinos oligonucleotides designed to abrogate *has3* splicing or translation. However, embryos injected with these anti-sense reagents exhibited developmental defects (S4 Fig), precluding functional studies at later developmental stages. As an alternative approach, it has been reported that 4-methylumbelliferone (4-MU) can selectively inhibit HA production by depleting the intracellular pool of UDP-GlcUA utilized by hyaluronan synthases [48]. 4-MU has also been used to suppress the accumulation of HA in damaged zebrafish heart tissue [50]. To see if 4-MU can inhibit zebrafish Has3-mediated HA synthesis, we treated HEK293 cells expressing zebrafish Has3 with varying concentrations of 4-MU (Fig 5; lanes 3–5). HEK293 cells overexpressing human HAS2 were also treated with this coumarin derivative to provide comparison controls (Fig 5; lanes 8–10). As expected, 4-MU blocked HA production mediated by either synthase in a dose-dependent manner and with a potency comparable to that described previously for *in vitro* studies (IC50 ~500 μM) [48,51,52].

**Fig 6:**
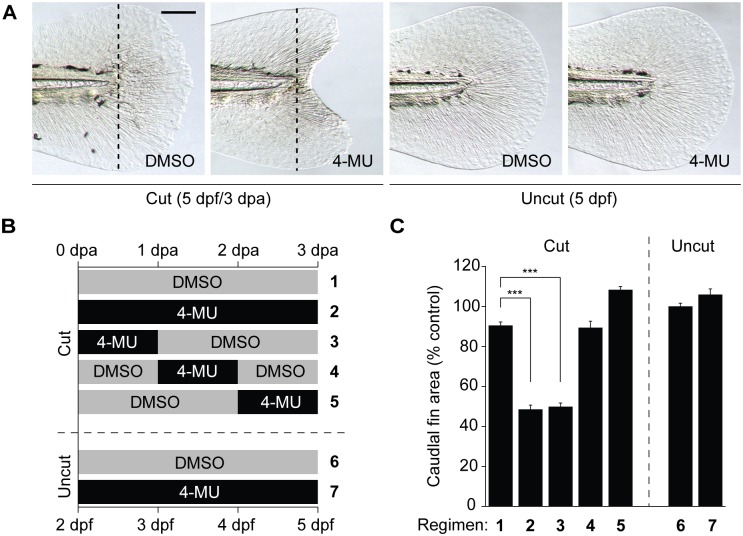
4-MU inhibits larval tail regeneration. (**A**) Representative micrographs of larval tails that were amputated at 2 dpf and then treated 0.5% DMSO or 150 μM 4-MU for 3 days. Dotted lines indicate the amputation plane, and micrographs of uncut larval tails subjected to the same inhibitor regimen are shown for comparison. Scale bar: 100 μm. (**B-C**) Time-course analysis of 4-MU action on larval tail regeneration. Caudal fin sizes at 5 dpf (3 dpa) after the indicated amputation and 4-MU treatment regimens. Data are the average caudal fin areas of 15 larvae ± s.e.m., normalized to the average fin size of uncut larvae treated with 0.5% DMSO. ***, *P* < 0.001.

**Fig 7:**
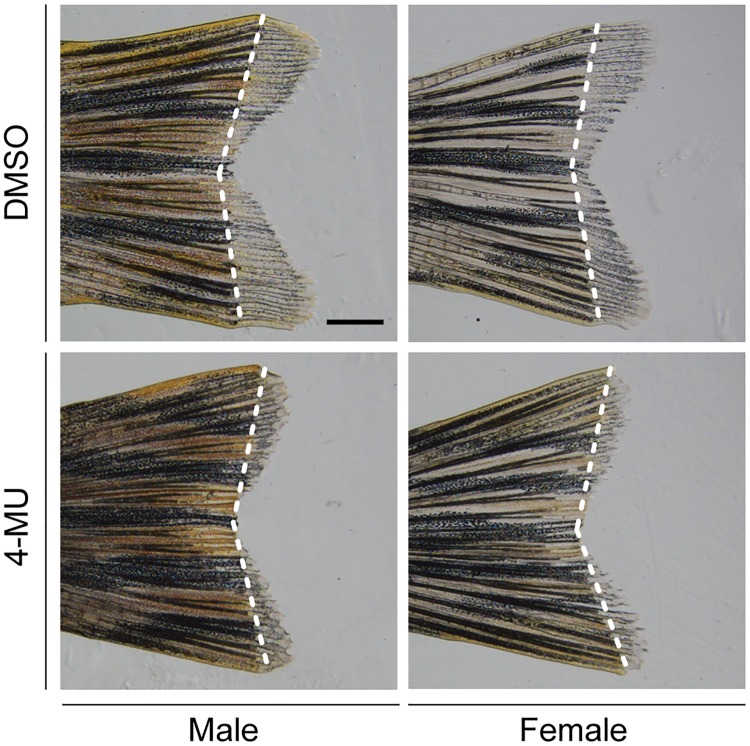
4-MU inhibits adult tail regeneration. Representative micrographs of adult tail fins that were amputated and then treated with 0.5% DMSO or 150 μM 4-MU for 7 days. White dotted lines indicate the amputation site. 10 adult zebrafish were analyzed for each experimental condition, and phenotypic descriptions were based on a penetrance of > 80%. Scale bar: 1 mm.

The ability of 4-MU to suppress zebrafish Has3-mediated HA production provided us with a valuable tool for studying the role of this glycosaminoglycan in larval tail regeneration. We observed that 150 μM 4-MU, a dose used previously for *in vivo* studies [40], induced a “concave” caudal fin morphology when applied from 0 to 3 dpa (2 to 5 dpf) (Fig 6A). However, the same 4-MU treatment protocol did not disrupt fish health or fin growth, consistent with the absence of *has1*, *has2*, or *has3* expression during larval tail development. To see if HA similarly promotes caudal fin regrowth at later life stages, we also treated adult fish with 150 μM 4-MU during the first week after tail amputation. The HA synthesis inhibitor significantly reduced tail regrowth in these fish (Fig 7), indicating that HA is essential for tail regeneration in both larvae and adult zebrafish.

We then investigated the temporal requirements of HA synthesis for larval tail regeneration by varying the time and duration of 4-MU treatment (Fig 6B). Exposure of tail-amputated larvae to 4-MU for the first 1 dpa caused regenerative defects by 3 dpa that were comparable to those induced by 3 days of continuous 4-MU treatment (Fig 6C). In contrast, 1-day exposures to 4-MU at later time points did not inhibit caudal fin regrowth. The temporal window of 4-MU sensitivity during tail regeneration therefore coincides with the timing of *has3* expression, consistent with their opposing effects on HA levels.

### 4-MU inhibits cell proliferation during tail regeneration

**Fig 8:**
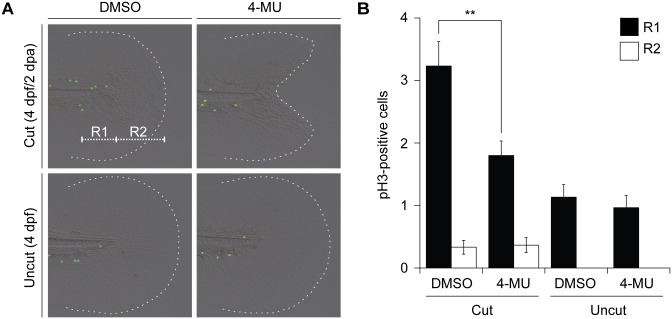
4-MU inhibits regenerative cell proliferation. (**A**) Mitotic cells in the larval tail after the indicated amputation and 4-MU treatment regimens, as visualized with anti-pH3 immunostaining at 2 dpa (4 dpf). R1 and R2 demarcate distinct regions within the larval tail, with R1 corresponding to a highly proliferative 100-μm-wide zone associated with tail regeneration. (**B**) Quantification of pH3-positive cells in the R1 and R2 regions under the indicated treatment conditions. Data are the average number of pH3-positive cells in 30 larval tails ± s.e.m. **, *P* < 0.01.

To understand the cellular mechanisms underlying these regeneration defects, we examined the effects of 4-MU on cell proliferation after tail amputation. We exposed tail-amputated larvae to 4-MU during the first 1 dpa, removed the HA synthesis inhibitor, and then assessed cell proliferation rates at 2 dpa by phosphorylated histone H3 (pH3) immunostaining. 4-MU treatment reduced the number of mitotic cells anterior to the amputation plane (Fig 8; region R1), a proximal region that has been previously shown to proliferate in response to caudal fin amputation [19]. In comparison, cell proliferation rates posterior to the amputation plane (Fig 8; region R2) were not affected by 4-MU. Cell division in uncut larval controls was also insensitive to 4-MU, matching the compound’s lack of an effect on larval growth and development. TUNEL analyses of larvae subjected to the same experimental conditions did not reveal significant numbers of apoptotic cells, with or without 4-MU treatment (data not shown). Collectively, these findings support a specific role for HA in regenerative cell proliferation.

### GSK3 inhibition rescues tail regeneration in 4-MU-treated larvae

4-MU has been previously reported to inhibit tail regeneration in *Xenopus* tadpoles, coinciding with a loss of Wnt/ß-catenin target gene expression within the regenerative bud [40]. Pharmacological and genetic inhibition of GSK3, a multifunctional kinase that primes ß-catenin for proteolytic degradation, was able to rescue tail regrowth in 4-MU-treated tadpoles, presumably by Wnt pathway activation [40]. We therefore investigated whether crosstalk between HA and GSK3 signaling also contributes to zebrafish tail regeneration. Tail-amputated zebrafish larvae were cultured in the presence of 4-MU, the GSK3 inhibitor BIO, or a combination of both compounds during the first 24 hours of regeneration, and the resulting morphological and cell proliferation phenotypes were assessed.

**Fig 9:**
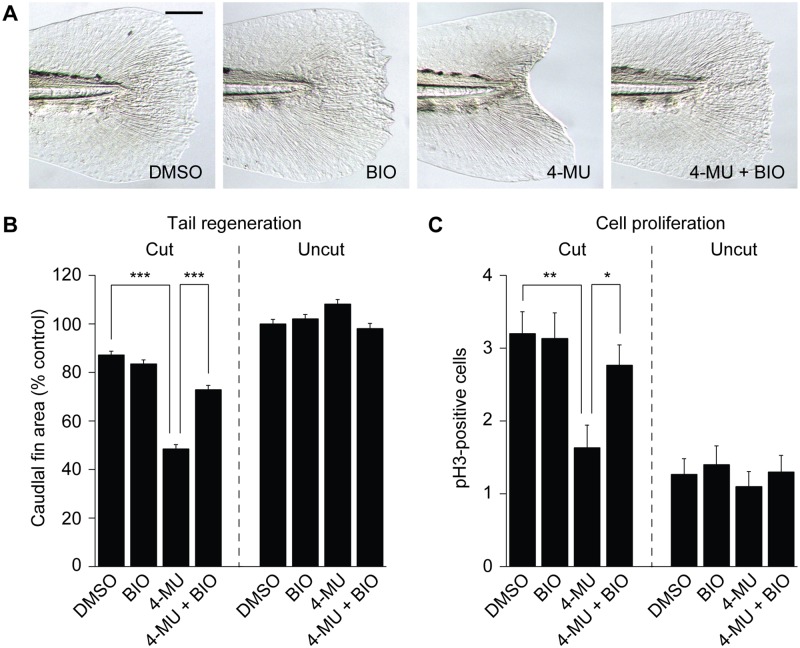
GSK3 inhibition rescues 4-MU-induced larval tail regeneration and cell proliferation defects. (**A**) Representative micrographs of larval tails that were amputated at 2 dpf and treated with 0.5% DMSO, 100 nM BIO, 150 μM 4-MU, or 150 μM 4-MU with 100 nM BIO for the next 24 hours. Caudal fins of 5-dpf (3-dpa) larvae are shown. Scale bar: 100 μm. (**B**) Caudal fin sizes at 5 dpf (3 dpa) for the indicated amputation and inhibitor treatment regimens (compound administration from 2 to 3 dpf). Data are the average caudal fin areas of 15 larvae ± s.e.m., normalized to the average fin size of uncut larvae treated with 0.5% DMSO. (**C**) Cell proliferation within the 4-dpf caudal fin in response to the indicated amputation and inhibitor treatment regimens. Data are the average number of pH3-positive cells in 30 larval tails ± s.e.m. (R1 + R2 regions; see Fig 9). *, *P* < 0.05; **, *P* < 0.01; ***, *P* < 0.001.

As before, 4-MU inhibited both cell proliferation anterior to the amputation plane at 4 dpf (2 dpa) and tail regrowth by 5 dpf (3 dpa), whereas BIO alone did not significantly affect either process (Fig 9A–9C). However, co-administration of BIO with 4-MU was able to reverse the anti-proliferative and anti-regenerative activities of the HA synthesis inhibitor (Fig 9A–9C). Similar rescues could be achieved with the structurally distinct GSK3 antagonists lithium chloride and SB216763, confirming the specificity of this effect (S5 Fig). The relative timing of 4-MU treatment and GSK3 inhibition was also critical for tail regrowth. In contrast to the rescue achieved by co-administration of 4-MU and BIO, caudal fin regeneration remained impaired when BIO dosing was initiated after the 24-hour 4-MU treatment (S6 Fig). Thus, HA and GSK3 signaling have opposing functions during the first day of tail regeneration, controlling proliferative cell populations that contribute to tail regrowth.

These signaling interactions appear to be conserved between *Xenopus* and zebrafish, and it has been proposed that HA acts through CD44 and HMMR receptors that are transiently expressed upon tadpole tail amputation [40]. Downstream signaling would then alleviate GSK3-mediated suppression of the Wnt pathway. In contrast to the tadpole system, however, we were not able to detect upregulation of *cd44*, *hmmr*, or Wnt pathway activity (S7 and S8 Figs) in tail-amputated zebrafish larvae, suggesting that HA and GSK3 might modulate caudal fin regeneration through other mechanisms.

### 4-MU and BIO differentially alter the expression of wound epithelium and blastema markers

**Fig 10:**
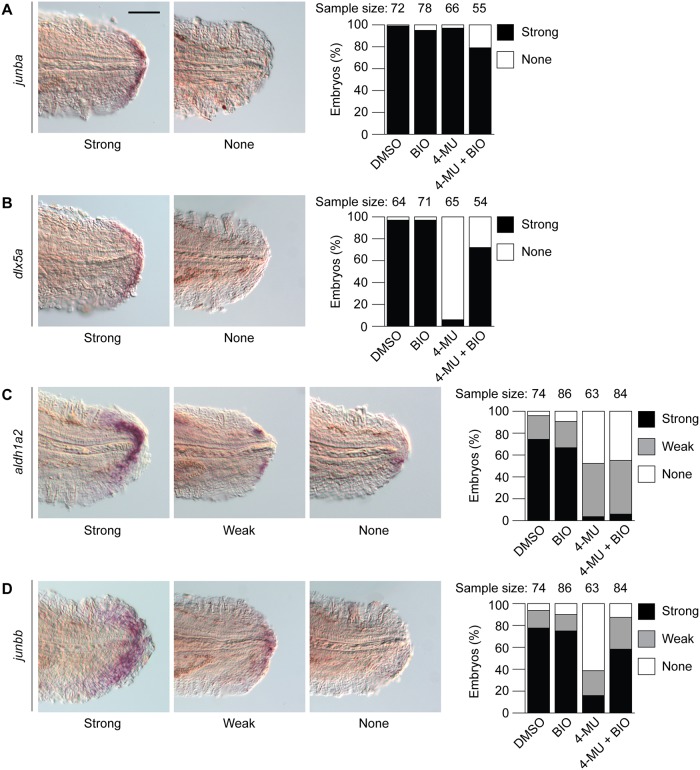
4-MU and GSK3 inhibition differentially control the expression of wound epithelium and blastema markers in larval tails. Effects of 0.5% DMSO, 100 nM BIO, 150 μM 4-MU, or 150 μM 4-MU with 100 nM BIO on *junba* (**A**), *dlx5a* (**B**), *aldh1a2* (**C**), and *junbb* (**D**) expression in 1-dpa (3-dpf) larval tails.

We concluded our studies by investigating the effects of 4-MU on specific cell populations within the regenerating tail. Previous studies have established critical roles for the wound epidermis and blastema in the regeneration of newt and axolotl limbs [53] and zebrafish tails [13]. We therefore examined whether pharmacological inhibition of HA synthesis disrupts formation of these tissues, using the transcription factor *dlx5a* and *junba* as wound epithelium markers [18,19,22,24–26,54] and *aldh1a2* and *junbb* as blastemal markers [15,22,24,25,44,54]. Using whole-mount *in situ* hybridization analyses, we found each of these genetic markers to be upregulated in the regenerating caudal fin by 1 dpa, and 4-MU treatment inhibited the expression of all but *junba* (Fig 10).

Since GSK3 inhibition can rescue the effects of 4-MU on larval tail regrowth, we also studied whether BIO could restore the expression of *dlx5a*, *aldh1a2*, and *junbb* in 4-MU-treated zebrafish. Although treatment of the tail-amputated larvae with BIO alone had no discernable effect on *dlx5a*, *aldh1a2*, or *junbb* expression levels (Fig 10), co-administration of this GSK3 inhibitor with 4-MU for the first 24 hours after tail amputation reestablished *dlx5a* and *junbb* transcription within the regenerative bud (Fig 10). Interestingly, *aldh1a2* expression remained suppressed under these conditions. Taken together, these results reveal a requirement for HA biosynthesis and signaling in establishing key cell populations within the wound epidermis and blastema of the regenerative bud, as well as an opposing role of GSK3 in this process. They also uncover differences between specific wound epithelium and blastema markers that could reflect contrasts in molecular and cellular functions.

## Discussion

By profiling the transcriptional changes that occur during larval tail regeneration in zebrafish, we have identified several potential regulators of this process, including 97 upregulated and 45 downregulated genes within the first day post amputation. Our findings complement previous microarray-based analyses of larval tail regrowth conducted by the Kawakami and Tanguay groups [24,25]. The Kawakami study discovered more than 200 upregulated and 20 downregulated genes (≥ 2-fold change at 16–24 hpa) [24], and the Tanguay study identified 131 upregulated and 58 downregulated genes (≤ 2.5-fold change at 1 dpa) [25]. A number of the genes identified in our transcriptome-wide analysis overlap with those reported by these two groups, including *aldh1a2*, *bcl2l10*, *fgf20a*, *fn1b*, *junba*, and *mmp9*, validating our general approach. However, the majority of genes identified in each larval tail regeneration study are unique to that particular survey. We speculate that these dissimilarities could arise from variations in the time and position of tail amputation, RNA extraction and amplification techniques, and microarray platforms used.

Among the novel findings in our study, we focus here on the role of HA in larval and adult tail regeneration in zebrafish. Tail amputation induces *has3* expression within six hours in zebrafish larvae, and *has1*, *has2*, *hyal2*, and *hyal4* are upregulated during adult tail regrowth. These transcriptional changes are consistent with recent reports that extracellular matrix rich in HA, tenascin C, and fibronectin forms during zebrafish heart and fin regeneration [55,56]. In principle, the differential expression of HA synthase isoforms and hyaluronidases in larval versus adult tails could reflect divergent or convergent mechanisms of HA-mediated regeneration. Previous cell-based and organismal studies have shown that the three HAS isoforms produce HA of differing sizes; HAS2 synthesizes HA polymers with molecular weights that exceed 2 MDa, and HAS1 and HAS3 generate forms that are approximately one-tenth the size [33,35,42]. Furthermore, HA length determines its biological function [33,36,37], and short (o-HA) and long (n-HA) forms of this glycosaminoglycan can have opposing activities [57]. Taken together, these results suggest that o-HA is specifically required for both larval and adult tail regeneration. In this model, such shorter polymers could be synthesized directly by Has3 in zebrafish larvae and through the degradation of Has1- and Has2-generated n-HA by hyaluronidases (Hyal2, Hyal4, and perhaps others) in adults. Consistent with this idea, *Has2* and *Hyal2* are upregulated in tail-amputated *Xenopus* tadpoles [40]. It has also been found that *Has2* overexpression inhibits tadpole tail regeneration, and this seemingly paradoxical outcome may reflect a shift in HA homeostasis toward a non-regenerative n-HA forms [40]. Thus, a requirement for o-HA in appendage regeneration could be conserved not only between developmental stages but also organismal species.

Importantly, we observe that the HA synthesis inhibitor 4-MU suppresses tail reformation in both larval and adult zebrafish, demonstrating an essential role for this glycosaminoglycan in the regenerative process. Taking advantage of the temporal control afforded by 4-MU and GSK3 inhibitors, we were able to establish the first 24 hours of larval tail regeneration as the critical period for HA action. Disrupting HA synthesis at later stages has no significant effect on caudal fin regrowth, and rescuing HA-induced defects by GSK3 inhibition requires addition of the kinase antagonist within the same time frame. Interestingly, early 4-MU treatment suppresses regenerative cell proliferation at later stages of tail regrowth. We hypothesize that HA signaling participates in early events within the tail wound epithelium that are required for establishing and/or maintaining a blastema-like zone within the regenerative bud. Accordingly, 4-MU suppresses the expression of *dlx5a* (though not *junba*) within the wound epidermis, indicating that at least some regenerative functions of this specialized tissue are lost. The HA synthesis inhibitor also abolishes expression of blastema markers such as *aldh1a2* and *junbb*. How HA generated within the wound epithelium might signal to blastemal cells is yet to be determined. These polysaccharides could diffuse from the epidermis to the underlying mesenchyme, thereby activating cognate receptors and downstream signaling pathways necessary for blastema formation and function. HA might also regulate blastema cell indirectly through other mechanisms, as several secreted growth factors have been found to mediate wound epidermis/blastema interactions that are essential for tissue regeneration [13,58].

Finally, our investigations provide insights into mechanisms that regulate and transduce HA signaling within the regenerative bud. Multiple developmental pathways are reactivated during the initial stages of caudal fin regrowth [5], and using small-molecule inhibitors, we were able to show their differential contributions to *has3* expression in tail-amputated zebrafish larvae. JNK and Notch signaling are primarily required for the onset of *has3* transcription, and FGF, PI3K, and TGFß signaling are also essential for its maintenance. How each of these pathways regulates *has3* expression remains unknown, but they could either promote the initial regenerative steps that lead to *has3* expression or play more direct roles in this process. In terms of downstream effectors of HA synthesis, we could detect expression of the HA receptors *cd44* and *hmmr* in amputated adult fins (S9 Fig), and overexpression of a dominant-negative form of CD44 inhibits tail regeneration in *Xenopus* tadpoles [40]. Similarly, increased expressions of *hmmr* and *cd44* were observed following ventricular resection in adult zebrafish, and morpholino-mediated knockdown of Hmmr impaired heart regeneration [50]. HA signaling through its cognate receptors CD44 or Hmmr is therefore likely required for these regenerative processes. However, we could not detect transcripts encoding these HA signaling proteins in corresponding larval tissues, suggesting that HA might interact with other receptors to promote regeneration at this life stage.

Intracellular signaling events downstream of HA may also appear to differ between regenerative processes. During adult zebrafish heart repair, a HA/Hmmr/FAK/Src pathway activates epicardial cell epithelial-mesenchymal transition (EMT) and migration [50], and HA functionally interact with GSK3 during tail regrowth in *Xenopus* tadpoles [40]. It appears that at least some aspects of HA response are conserved between *Xenopus* and zebrafish tail regeneration. As previously reported in the tadpole study [40], we observed that GSK3 inhibition rescues the tail regeneration defects caused by 4-MU, as well as the coincident effects of this HA synthesis inhibitor on regenerative cell proliferation. The precise mechanisms of HA/GSK3 crosstalk remain unknown, but one possibility is that HA promotes tail regrowth by activating intracellular pathways that suppress GSK3 activity. Indeed, pharmacological inhibition of one such pathway, PI3K/Akt signaling [23,43], also blocks larval tail regeneration, and GSK3 inhibitors can reverse these defects (S10 Fig).

Although it has been proposed that HA-mediated suppression of GSK3 activates ß-catenin-dependent Wnt target genes in *Xenopus* tadpoles [40], we did not observe Wnt pathway activation in tail-amputated zebrafish larvae, even when treated with GSK3 inhibitors (see S8 Fig). Given the pleiotropic functions of GSK3 [59], this kinase may control larvae tail regeneration through ß-catenin-independent cellular functions [60]. Interestingly, GSK3 inhibition rescues the expression of *junbb* but not *aldh1a2* in amputated larval tails treated with 4-MU. GSK3 may therefore act downstream of Aldh1a2 in regulating blastema function, or alternatively, the two blastemal markers could label distinct subsets of cells with differential responses to GSK3. This latter possibility is evidenced by the broad range of expression patterns observed for upregulated genes within the regenerating tail (see S1 and S2 Figs). Indeed, HA may play important roles in coordinating these diverse cell populations as they re-establish the caudal fin.

## Supporting information

S1 Fig Genes expressed in distal cells during larval tail regeneration. (**A-E**) Expression patterns of selected genes transcribed in the regenerative bud at 1 dpa (3 dpf), as determined by whole-mount *in situ* hybridization. (**A’-E’**) Equivalently stained uncut controls. At least 30 larvae were analyzed for each experimental condition, and phenotypic descriptions were based on a penetrance of > 80%. Scale bar: 100 μm.
(TIF)

S2 Fig Genes expressed in blastema-like cells during larval tail regeneration. (**A-D**) Expression patterns of selected genes transcribed in the regenerative bud at 1 dpa (3 dpf), as determined by whole-mount *in situ* hybridization. (**A’-D’**) Equivalently stained uncut controls. At least 30 larvae were analyzed for each experimental condition, and phenotypic descriptions were based on a penetrance of > 80%. Scale bar: 100 μm.
(TIF)

S3 Fig Inhibition of larval tail regeneration by pathway-specific antagonists. Representative micrographs of larval tails that were amputated at 2 dpf and then treated with the following signaling pathway inhibitors for 3 days: (**A**) 0.5% DMSO; (**B**) 75 μM PD173074 (FGF); (**C**) 10 μM LY294002 (PI3K); (**D**) 50 μM SB431542 (TGFß); (**E**) 5 μM SP600125 (JNK); (**F**) 50 μM DAPT (Notch); (**G**) 100 μM cyclopamine (Hh); or (**H**) 50 μM dorsomorphin (BMP). At least 30 larvae were analyzed for each experimental condition, and phenotypic descriptions were based on a penetrance of > 80%. Scale bar: 100 μm.
(TIF)

S4 Fig Developmental defects in *has3* morphants. Representative micrographs of 28-hpf embryos injected with morpholino oligonucleotides targeting either the *has3* translational start site (**A**; ATG-MO, 10 ng/embryo) or the *has3* intron 2-exon 3 splice junction (**B**; i2e3-MO, 16 ng/embryo). Scale bar: 200 μm. (**C**) Confirmation of *has3* i2e3-MO-dependent target mRNA missplicing by RT-PCR.
(TIF)

S5 Fig GSK3 inhibitors lithium chloride and SB216763 also rescue 4-MU-induced larval tail regeneration defects. (**A**) Chemical structures of BIO and SB216763. (**B**) Caudal fin sizes at 5 dpf (3 dpa) after amputation at 2 dpf and treatment with designated inhibitors for 1 day. Compound concentrations: 4-MU, 150 μM; LiCl, 150 μM; SB216753, 50 μM. Data are the average caudal fin areas of 15 larvae ± s.e.m., normalized to the average fin size of uncut larvae treated with 0.5% DMSO. ***, *P* < 0.001.
(TIF)

S6 Fig Concurrent GSK3 inhibition is required to rescue 4-MU-induced larval tail regeneration defects. Caudal fin sizes at 5 dpf (3 dpa) after amputation at 2 dpf and the indicated inhibitor treatment regimens. Data are the average caudal fin areas of 15 larvae ± s.e.m., normalized to the average fin size of uncut larvae treated with 0.5% DMSO (inhibitor regimen 5). ***, *P* < 0.001; n.s. = not significant.
(TIF)

S7 Fig Zebrafish *cd44* and *hmmr* are not visible upregulated during larval tail regeneration. Whole-mount *in situ* hybridization of 1-dpa (3-dpf) larval tails with riboprobes for *cd44* (**A**) or *hmmr* (**B**) at 1 dpa. (**A’** and **B’**) Equivalently stained uncut controls. At least 30 larvae were analyzed for each experimental condition, and phenotypic descriptions were based on a penetrance of > 80%. Scale bar: 100 μm.
(TIF)

S8 Fig Wnt pathway activity is not upregulated during larval tail regeneration or modulated by 4-MU or GSK3 inhibitors. Whole-mount analysis of *GFP* expression in both wild type (**A-A’**) and *Tg*(*top*:*GFP*) embryos (**B-E** and **B’-E’**) at 1 dpa (3 dpf), following treatments with DMSO, 150 μM 4-MU, 100 nM BIO, or 50 μM SB216763 from 2 to 3 dpf. The *Tg*(*top*:*GFP*) embryos express a destabilized form of GFP under control of a minimal cFos promoter with four TCF/LEF binding sites, providing a dynamic readout of Wnt pathway state. At least 30 larvae were analyzed for each experimental condition, and phenotypic descriptions were based on a penetrance of > 80%. Scale bar: 300 μM.
(TIF)

S9 Fig Hyaluronic acid receptors are expressed during adult zebrafish tail regeneration. Expression patterns of *cd44* (**A**) and *hmmr* (**B**) in adult tails at 2 dpa. 10 adult zebrafish were analyzed for each experimental condition, and phenotypic descriptions were based on a penetrance of > 80%. Scale bar: 300 μm.
(TIF)

S10 Fig GSK3 inhibition rescues LY294002-induced tail regeneration defects. (**A**) Representative micrographs of 5-dpf larval tails that were amputated at 2 dpf and treated with 0.5% DMSO, 10 μM LY294002, or 10 μM LY294002 + 100 nM BIO for the next 24 hours. Scale bar: 100 μm. (**B**) Caudal fin sizes at 5 dpf (3 dpa) for the indicated amputation and inhibitor treatment regimens (compound administration from 2 to 3 dpf). Data are the average caudal fin areas of 15 larvae ± s.e.m., normalized to the average fin size of uncut larvae treated with 0.5% DMSO. ***, *P* < 0.001.
(TIF)

S1 Table PCR primers used to amplify gene-specific cDNAs for the *in vitro* transcription of digoxigenin-labeled RNA probes. (PDF)

S2 Table Genes that exhibited ≥ 1.5 fold change in expression after zebrafish larval tail amputation (false discovery rate < 0.1; microarray hits ranked according to fold change). (PDF)
