# Supplementary figures and images for: Hyaluronic acid synthesis is required for zebrafish tail fin regeneration
Source: PLoS One. 2017 Feb 16;12(2):e0171898. doi: 10.1371/journal.pone.0171898 (PMC5313160; doi:10.1371/journal.pone.0171898)

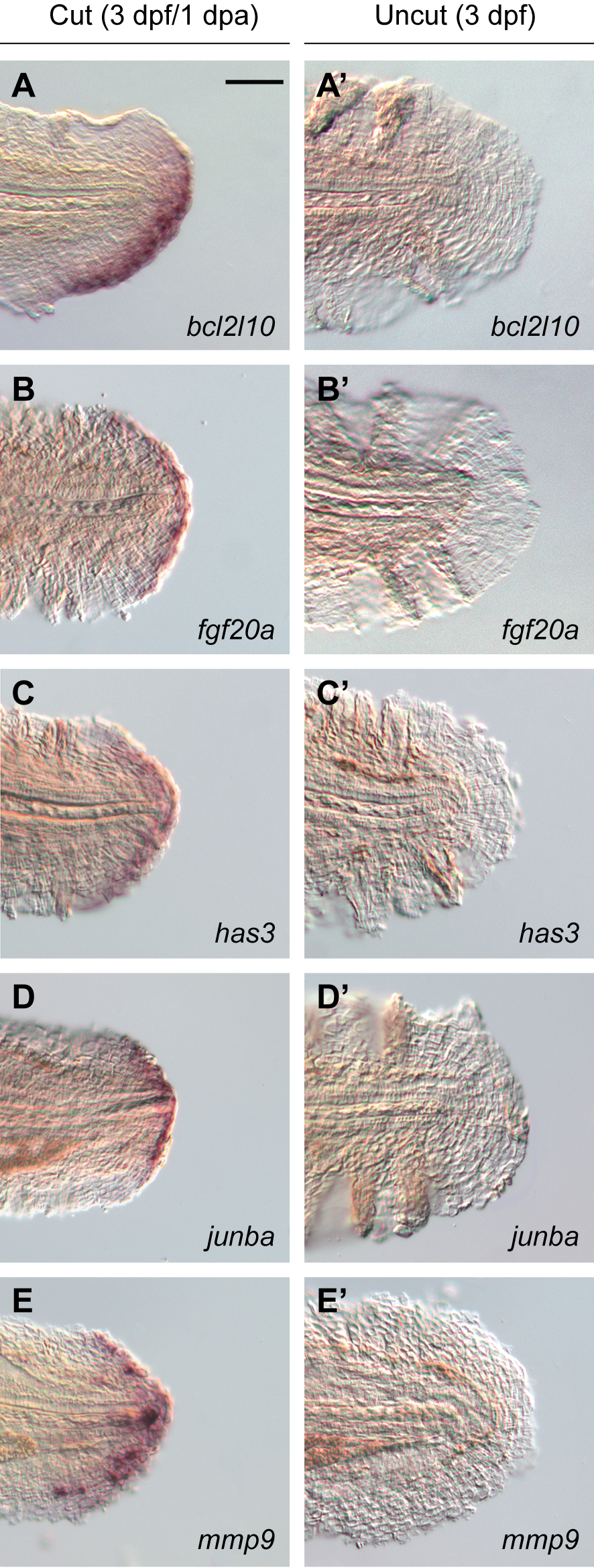

Supplement: S1 Fig — (A-E) Expression patterns of selected genes transcribed in the regenerative bud at 1 dpa (3 dpf), as determined by whole-mount in situ hybridization. (A’-E’) Equivalently stained uncut controls. At least 30 larvae were analyzed for each experimental condition, and phenotypic descriptions were based on a penetrance of > 80%. Scale bar: 100 μm. (TIF) [file pone.0171898.s001.tif]

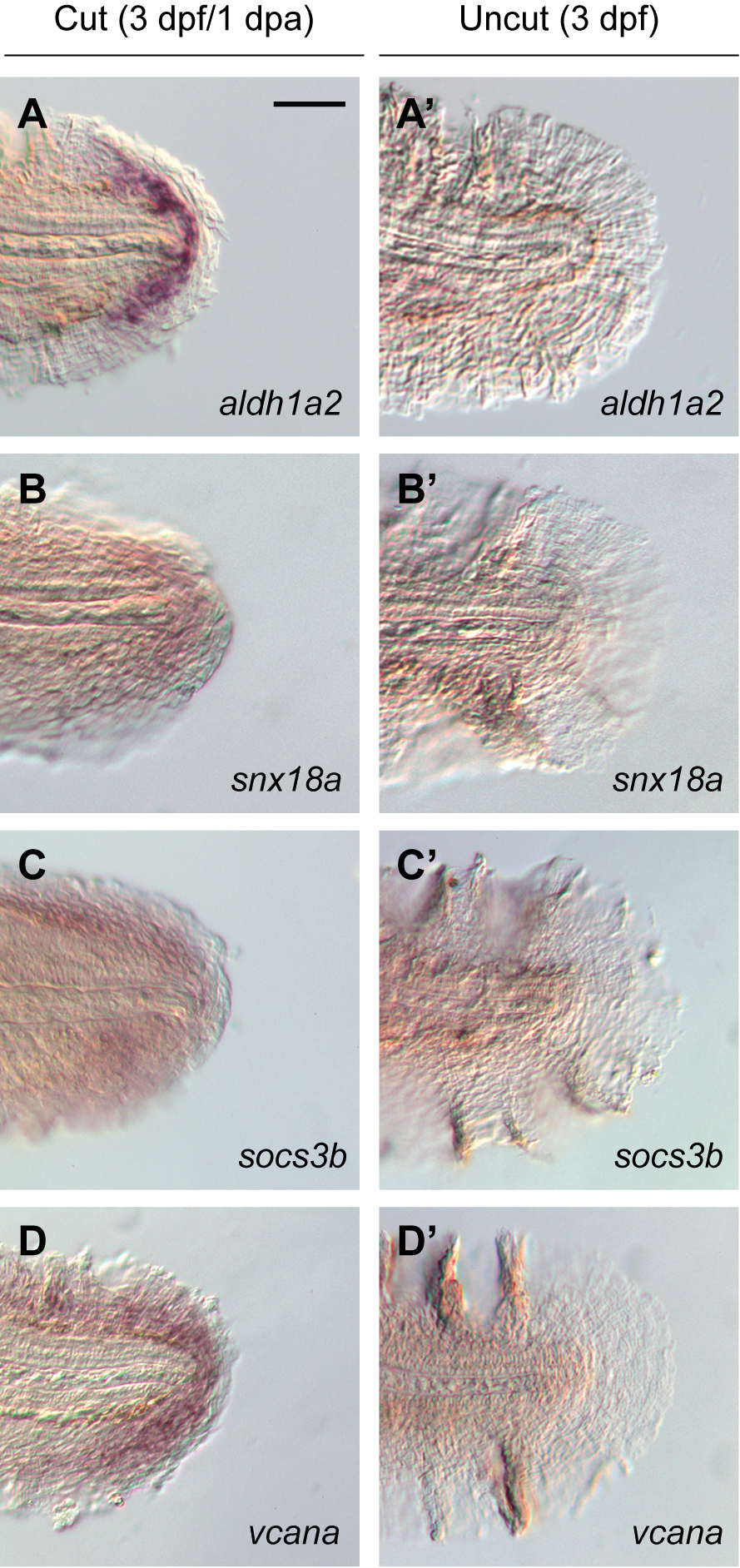

Supplement: S2 Fig — (A-D) Expression patterns of selected genes transcribed in the regenerative bud at 1 dpa (3 dpf), as determined by whole-mount in situ hybridization. (A’-D’) Equivalently stained uncut controls. At least 30 larvae were analyzed for each experimental condition, and phenotypic descriptions were based on a penetrance of > 80%. Scale bar: 100 μm. (TIF) [file pone.0171898.s002.tif]

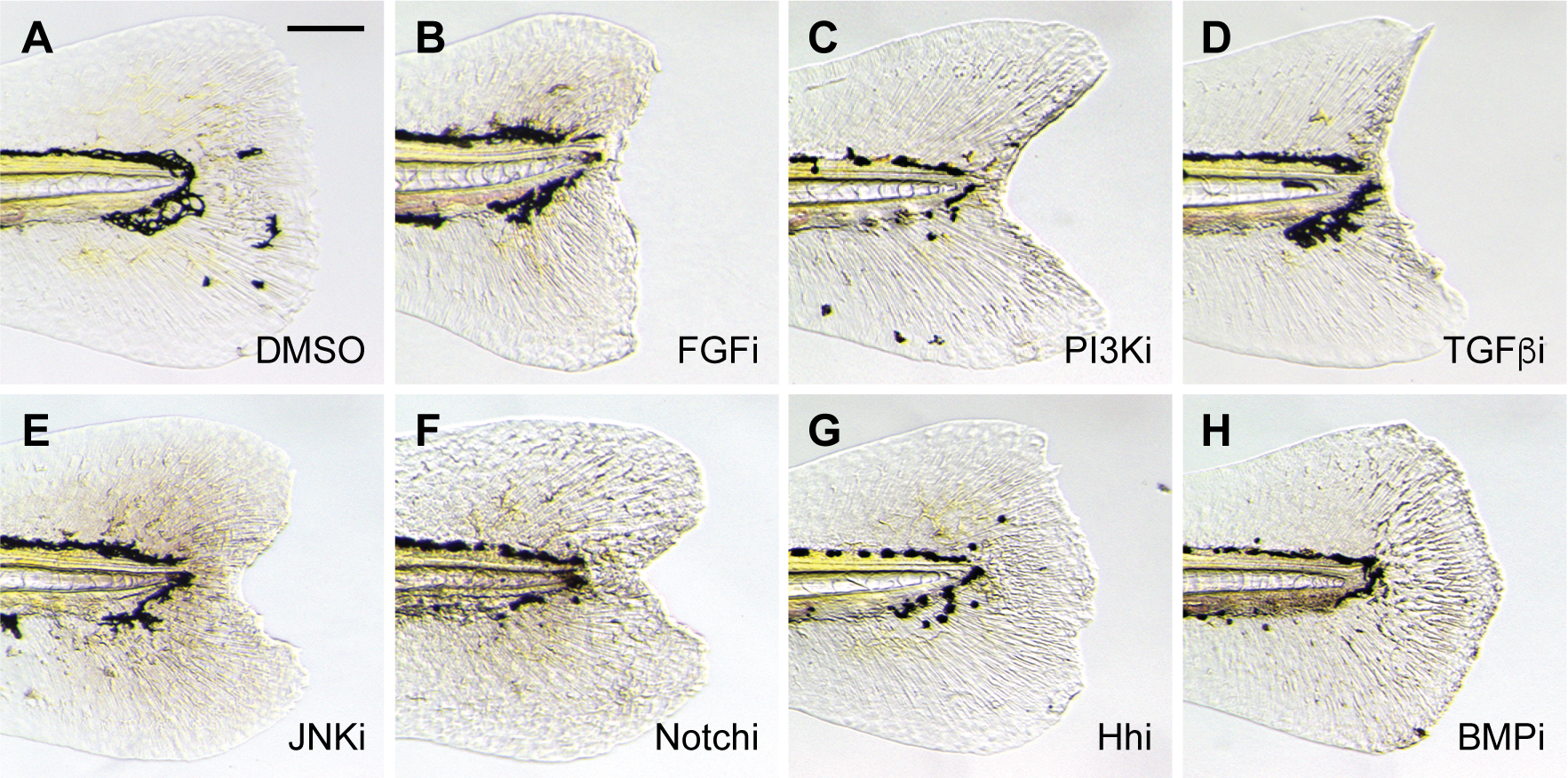

Supplement: S3 Fig — Representative micrographs of larval tails that were amputated at 2 dpf and then treated with the following signaling pathway inhibitors for 3 days: (A) 0.5% DMSO; (B) 75 μM PD173074 (FGF); (C) 10 μM LY294002 (PI3K); (D) 50 μM SB431542 (TGFß); (E) 5 μM SP600125 (JNK); (F) 50 μM DAPT (Notch); (G) 100 μM cyclopamine (Hh); or (H) 50 μM dorsomorphin (BMP). At least 30 larvae were analyzed for each experimental condition, and phenotypic descriptions were based on a penetrance of > 80%. Scale bar: 100 μm. (TIF) [file pone.0171898.s003.tif]

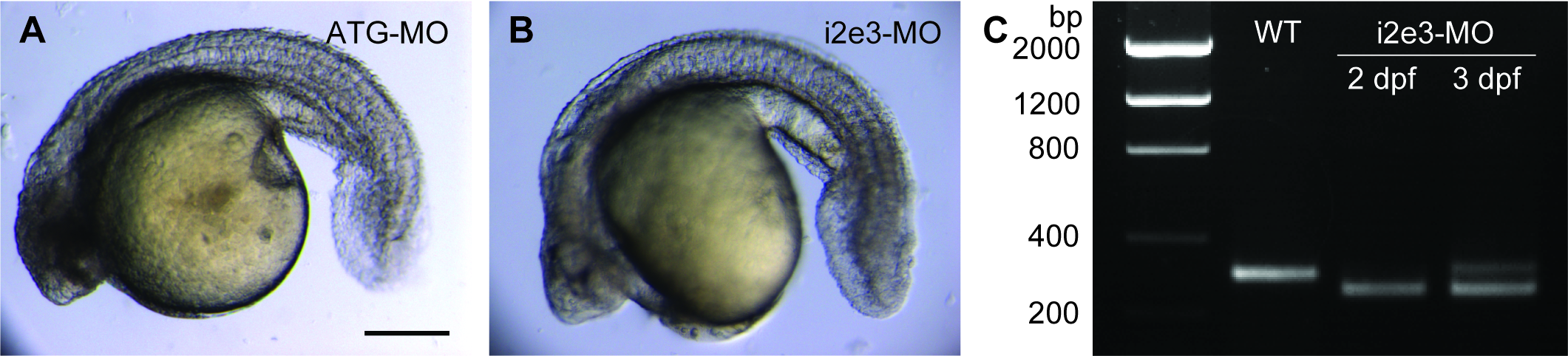

Supplement: S4 Fig — Representative micrographs of 28-hpf embryos injected with morpholino oligonucleotides targeting either the has3 translational start site (A; ATG-MO, 10 ng/embryo) or the has3 intron 2-exon 3 splice junction (B; i2e3-MO, 16 ng/embryo). Scale bar: 200 μm. (C) Confirmation of has3 i2e3-MO-dependent target mRNA missplicing by RT-PCR. (TIF) [file pone.0171898.s004.tif]

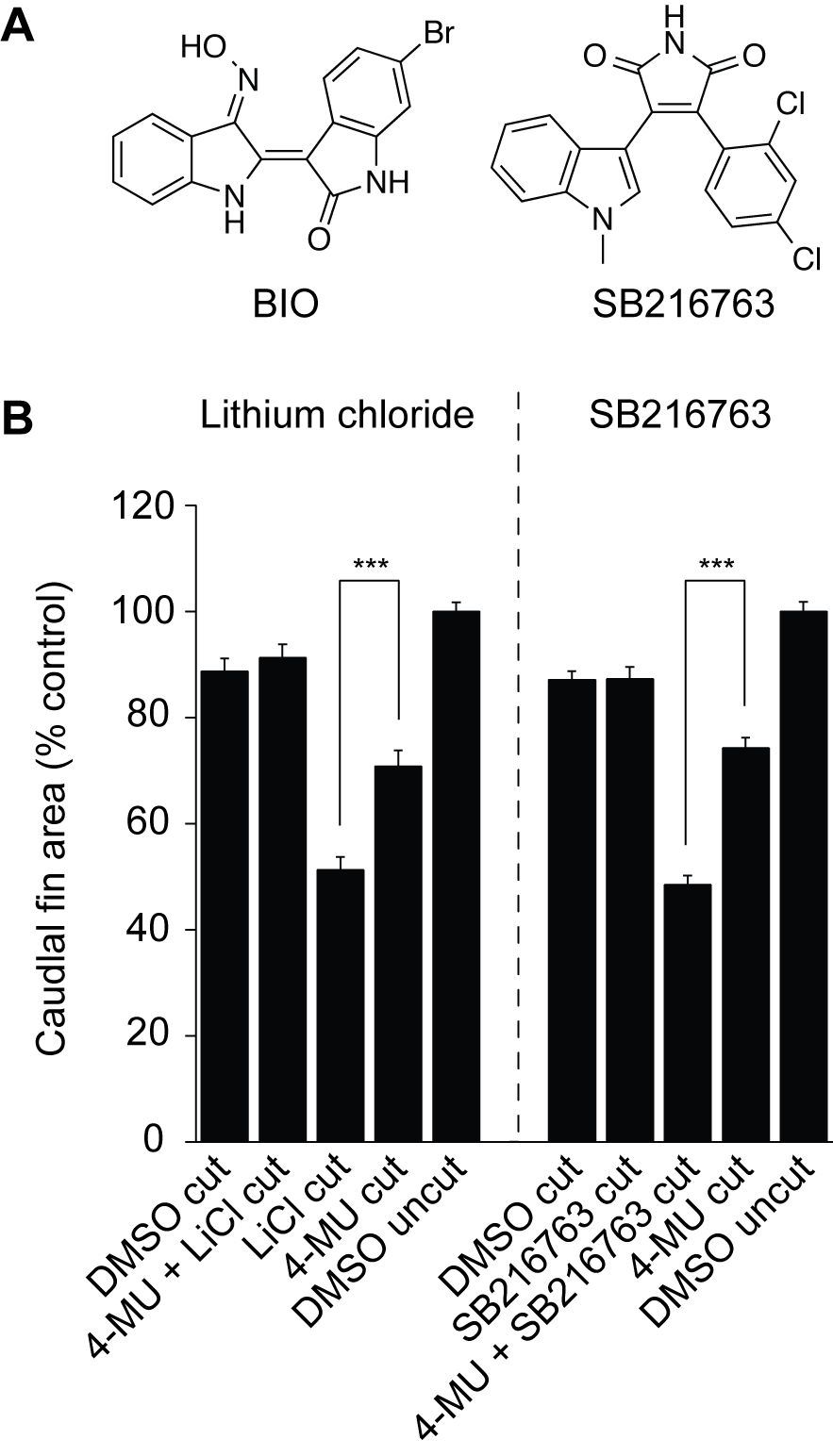

Supplement: S5 Fig — (A) Chemical structures of BIO and SB216763. (B) Caudal fin sizes at 5 dpf (3 dpa) after amputation at 2 dpf and treatment with designated inhibitors for 1 day. Compound concentrations: 4-MU, 150 μM; LiCl, 150 μM; SB216753, 50 μM. Data are the average caudal fin areas of 15 larvae ± s.e.m., normalized to the average fin size of uncut larvae treated with 0.5% DMSO. ***, P < 0.001. (TIF) [file pone.0171898.s005.tif]

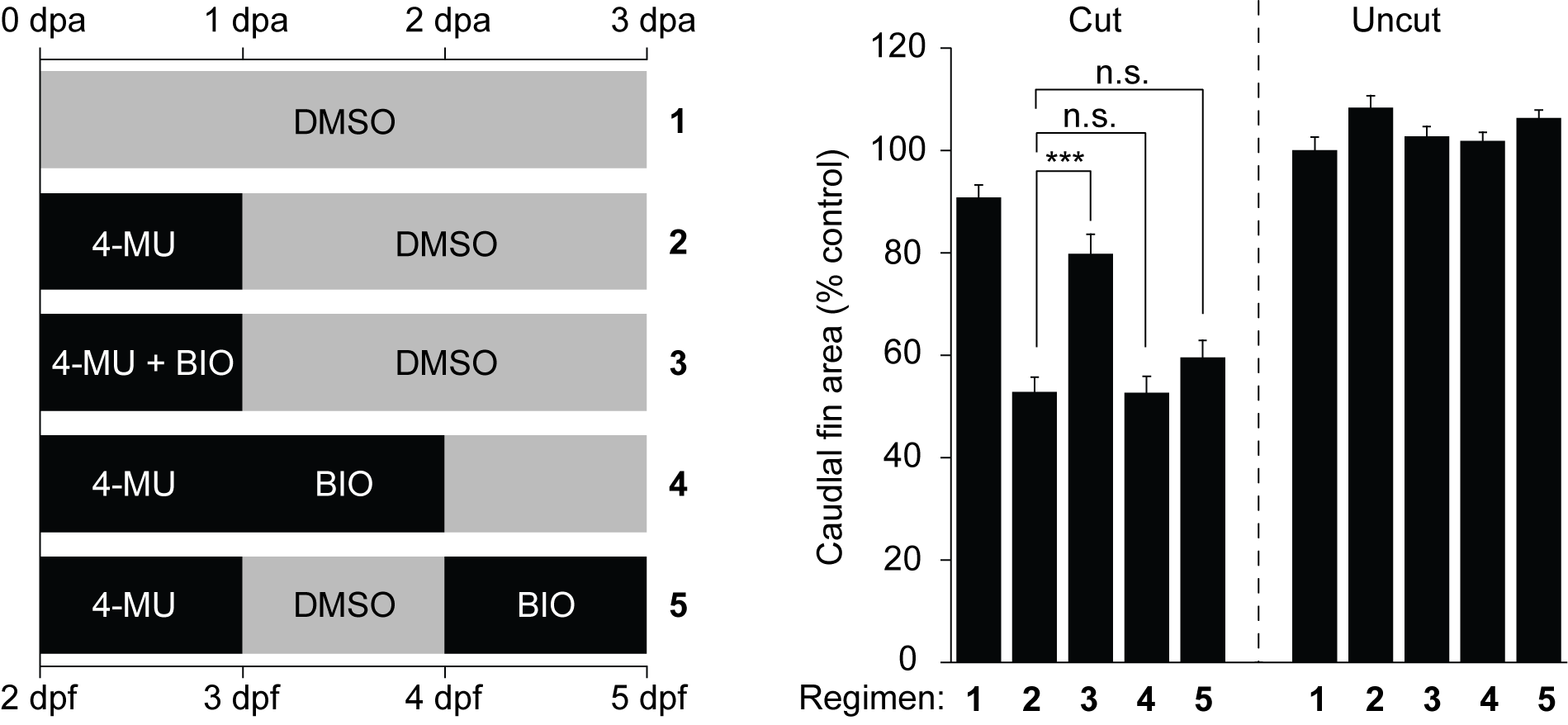

Supplement: S6 Fig — Caudal fin sizes at 5 dpf (3 dpa) after amputation at 2 dpf and the indicated inhibitor treatment regimens. Data are the average caudal fin areas of 15 larvae ± s.e.m., normalized to the average fin size of uncut larvae treated with 0.5% DMSO (inhibitor regimen 5). ***, P < 0.001; n.s. = not significant. (TIF) [file pone.0171898.s006.tif]

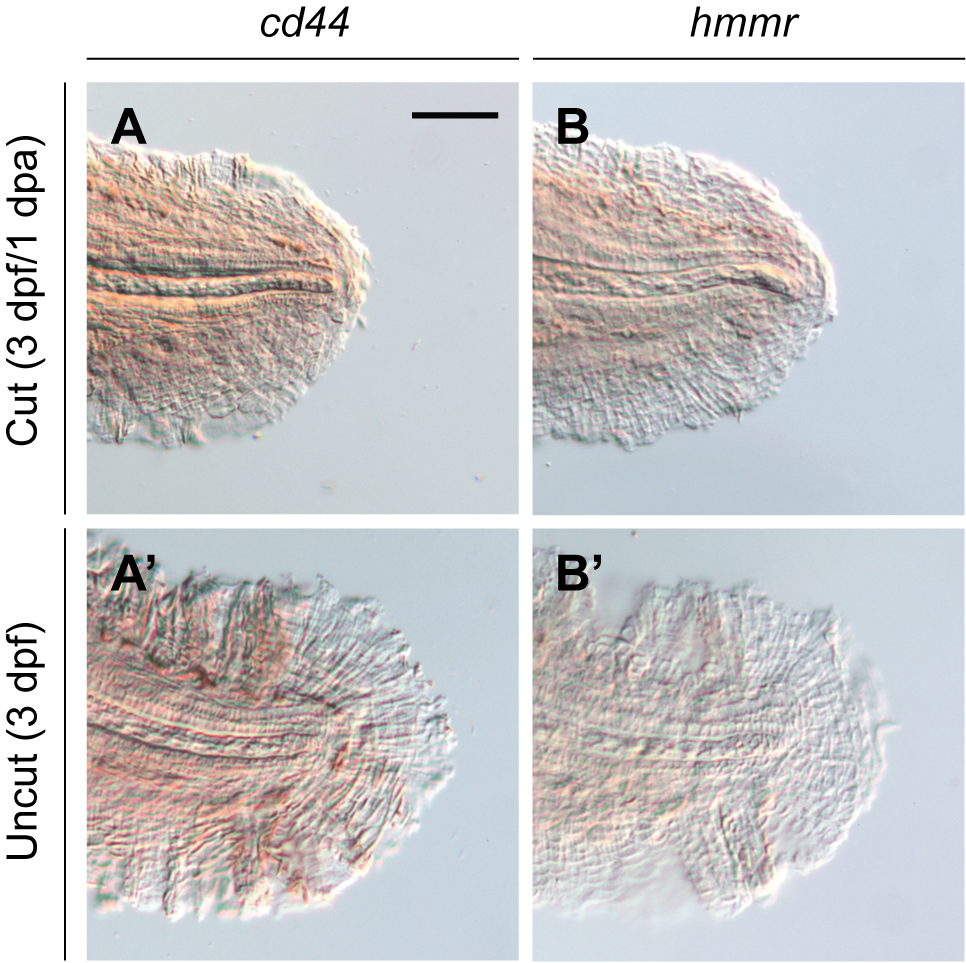

Supplement: S7 Fig — Whole-mount in situ hybridization of 1-dpa (3-dpf) larval tails with riboprobes for cd44 (A) or hmmr (B) at 1 dpa. (A’ and B’) Equivalently stained uncut controls. At least 30 larvae were analyzed for each experimental condition, and phenotypic descriptions were based on a penetrance of > 80%. Scale bar: 100 μm. (TIF) [file pone.0171898.s007.tif]

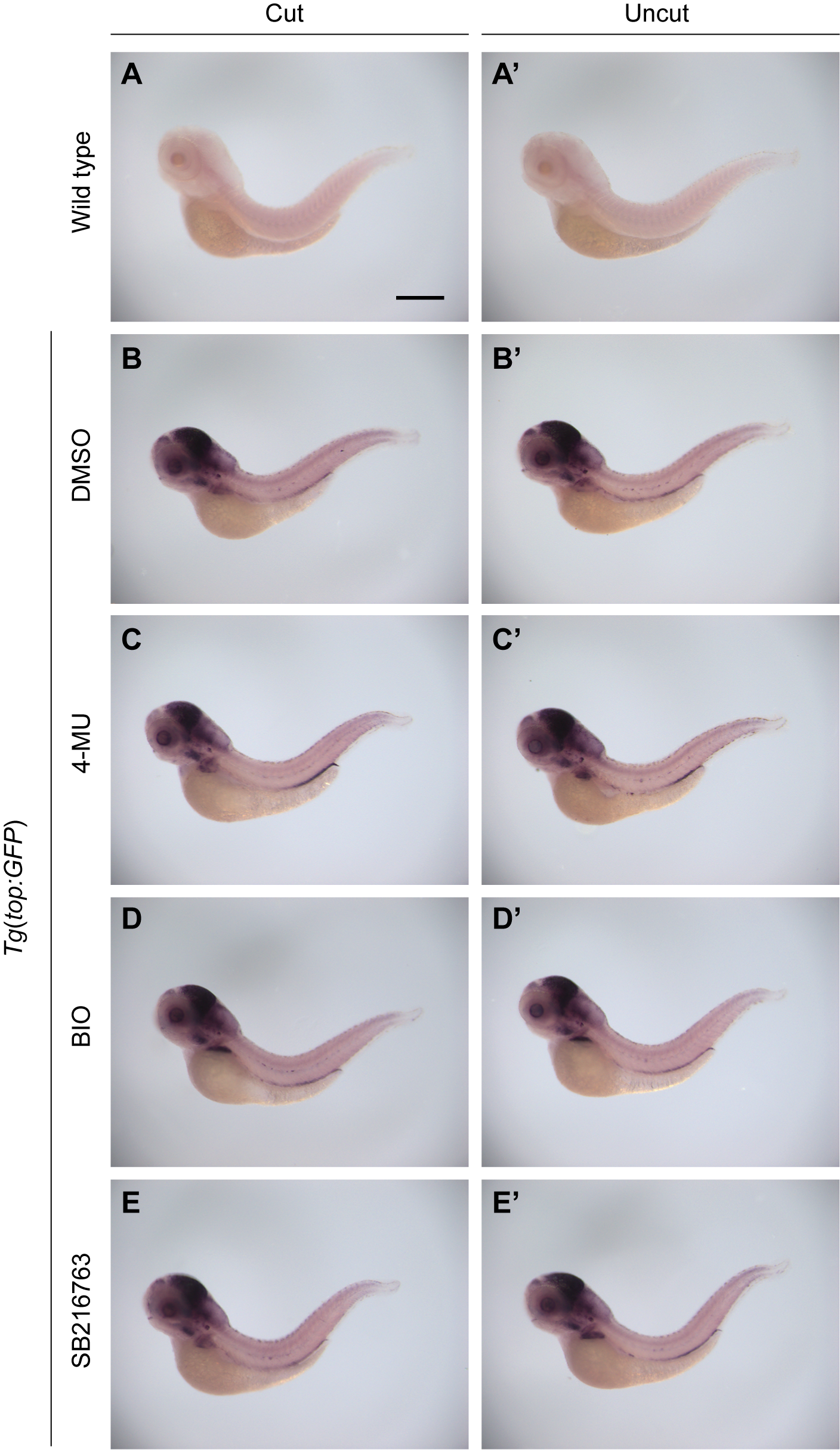

Supplement: S8 Fig — Whole-mount analysis of GFP expression in both wild type (A-A’) and Tg(top:GFP) embryos (B-E and B’-E’) at 1 dpa (3 dpf), following treatments with DMSO, 150 μM 4-MU, 100 nM BIO, or 50 μM SB216763 from 2 to 3 dpf. The Tg(top:GFP) embryos express a destabilized form of GFP under control of a minimal cFos promoter with four TCF/LEF binding sites, providing a dynamic readout of Wnt pathway state. At least 30 larvae were analyzed for each experimental condition, and phenotypic descriptions were based on a penetrance of > 80%. Scale bar: 300 μM. (TIF) [file pone.0171898.s008.tif]

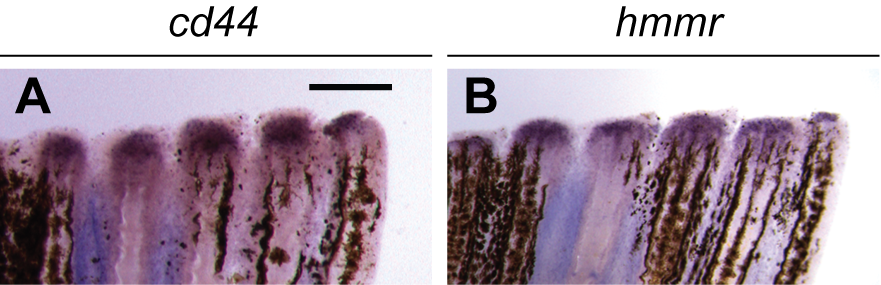

Supplement: S9 Fig — Expression patterns of cd44 (A) and hmmr (B) in adult tails at 2 dpa. 10 adult zebrafish were analyzed for each experimental condition, and phenotypic descriptions were based on a penetrance of > 80%. Scale bar: 300 μm. (TIF) [file pone.0171898.s009.tif]

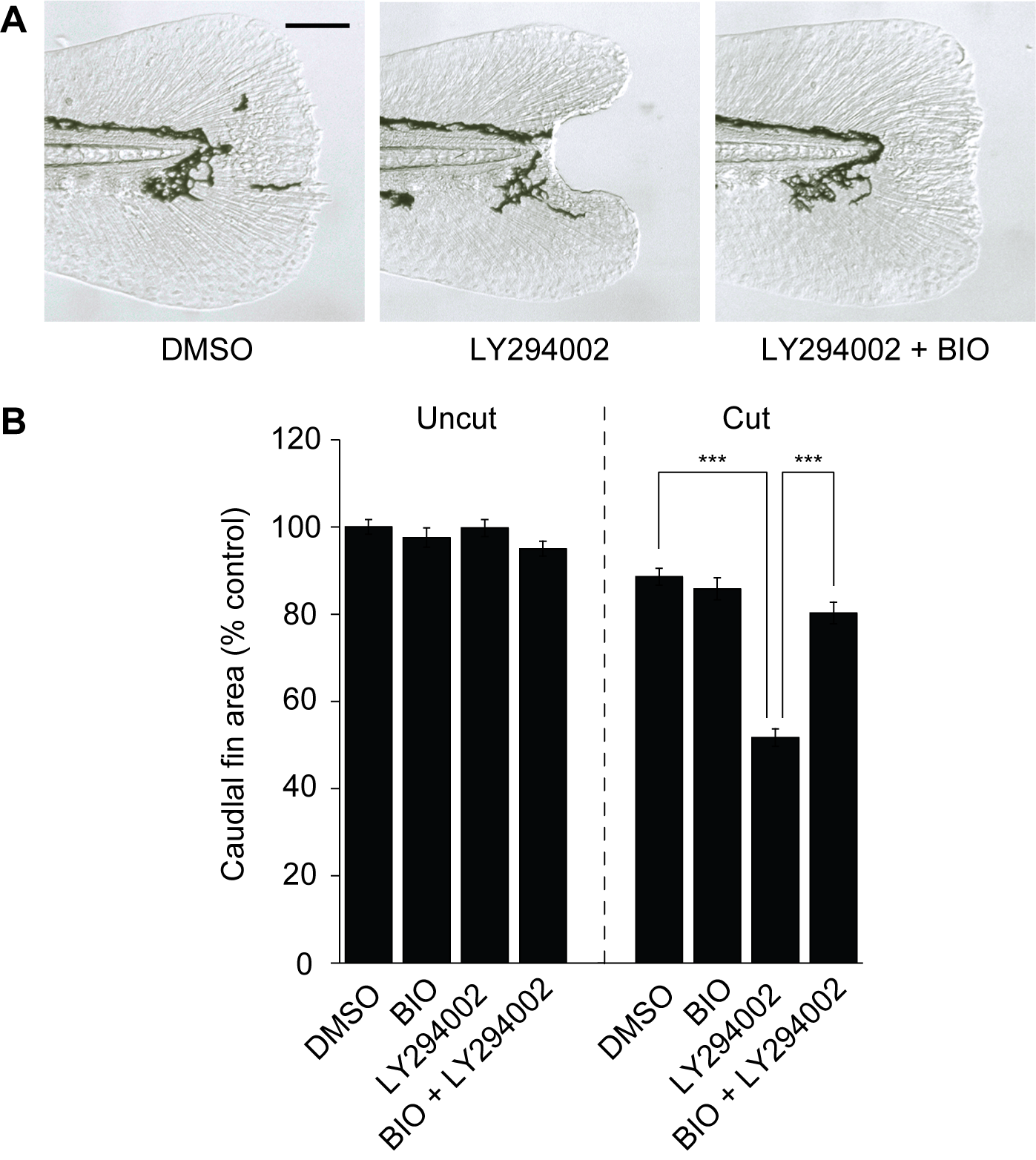

Supplement: S10 Fig — (A) Representative micrographs of 5-dpf larval tails that were amputated at 2 dpf and treated with 0.5% DMSO, 10 μM LY294002, or 10 μM LY294002 + 100 nM BIO for the next 24 hours. Scale bar: 100 μm. (B) Caudal fin sizes at 5 dpf (3 dpa) for the indicated amputation and inhibitor treatment regimens (compound administration from 2 to 3 dpf). Data are the average caudal fin areas of 15 larvae ± s.e.m., normalized to the average fin size of uncut larvae treated with 0.5% DMSO. ***, P < 0.001. (TIF) [file pone.0171898.s010.tif]
