# Supplementary material for: Hyaluronic acid synthesis is required for zebrafish tail fin regeneration
Source: PLoS One. 2017 Feb 16;12(2):e0171898. doi: 10.1371/journal.pone.0171898 (PMC5313160; doi:10.1371/journal.pone.0171898)
Supplement: S1 Table — (PDF) [file pone.0171898.s011.pdf]

S1 Table. PCR primers used to amplify gene-specific cDNAs for the *in vitro* transcription of digoxigenin-labeled RNA probes.

Gene Symbol	Primer Sequences (5' to 3')
aldh1a2	F: ACTGCCAGGAGAGGTGAAGA<br>R: CGTAATACGACTCACTATAGGGGCCTGTTCTAATGCCAGCTC
bcl2l10	F: GCACAGAACCAAATTCCGAT<br>R: CGTAATACGACTCACTATAGGGGAAATCGCTTGGAACCAAAA
cd44	F: GTCTGCATTGCTGTCCTCCT<br>R: CGTAATACGACTCACTATAGGGTGCTTCTGTAGGGCTGTGTG
dlx5a	F: AGAGACGCTTGACGCTTGCAC<br>R: CGTAATACGACTCACTATAGGGAACGTGCCGGGTGCCTGAAG
fgf20a	F: ATGGGTGCAGTCGGCGAGC<br>R: CGTAATACGACTCACTATAGGGAAGCTCAGGAACTCGCTCTGGA
fn1b	F: TCGTGGACTGGAGCCAGGCAT<br>R: CGTAATACGACTCACTATAGGGACGCAATGCTTCCACCCGCA
has1	F: TGTGTCTCTGGTCCGAGCCTT<br>R: CGTAATACGACTCACTATAGGGTCCCTCTTTCCTCCCCATTTCTGC
has2	F: ACTTCAACCTGTGCGCTTGGGG<br>R: CGTAATACGACTCACTATAGGGGCCGGGTCCAGCATGGTGTC
has3	F: CACACAGAGCAGCACCATCT<br>R: CGTAATACGACTCACTATAGGGGATTGAGCCAACGCAGGTAT
hmmr	F: CATGGAGCGCCGGGTGTCTG<br>R: CGTAATACGACTCACTATAGGGGCTTCTGCTGGCCCTGCTGT
hyal2	F: AGAGTGGCGGCCCCTTTGGA<br>R: CGTAATACGACTCACTATAGGGCGCCGCTCGATTCCCGTTGT
hyal3	F: CCGCCTGGGCCTCTACCCTT<br>R: CGTAATACGACTCACTATAGGGGCAGTTCGTCACTCCGGCCC
hyal4	F: GCACAACTGTTCCAAAGTGAAGGCA<br>R: CGTAATACGACTCACTATAGGGTGATGTGAGTCTGGGGTGGGGT
hyal6	F: AAACTGGCGACCGCAGTGGG<br>R: CGTAATACGACTCACTATAGGGAGGCTCTGCCATCTGGGGCA
junba	F: ACGGAGAGAACTTGCGGACT<br>R: CGTAATACGACTCACTATAGGGCTGCTCTTCCAGTGTTTCCC
junbb	F: ACGTGACCGAGCCGCCCTAT<br>R: CGTAATACGACTCACTATAGGGGGCCGCAGTGTCCGTTCTC
mmp9	F: CCCTTGGCTCTGGACCAGCCAT<br>R: CGTAATACGACTCACTATAGGGAGTCGCTTGGTAGGCCGAGCT
snx18a	F: CCCTCTGGCTACAGCATCTC<br>R: CGTAATACGACTCACTATAGGGAGTTGGTGAGGTGTCCTTGG
socs3b	F: GCTCCCATCTAGCCCTCTCT<br>R: CGTAATACGACTCACTATAGGGCGGTCATCAGACCAGACCTT
vcana	F: TGAAAACTGGAGACCGAACC<br>R: CGTAATACGACTCACTATAGGGAGGAAATCCTGGGAGAGTCC

The T7 promoter sequence is indicated in blue.
