# Supplementary material for: Hyaluronic acid synthesis is required for zebrafish tail fin regeneration
Source: PLoS One. 2017 Feb 16;12(2):e0171898. doi: 10.1371/journal.pone.0171898 (PMC5313160; doi:10.1371/journal.pone.0171898)
Supplement: S2 Table — (PDF) [file pone.0171898.s012.pdf]

S2 Table. **Genes that exhibited ≥ 1.5-fold change in expression after zebrafish larval tail amputation** (false discovery rate < 0.1; microarray hits ranked according to fold change).

Entry	Gene Symbol	Gene Description	Fold Change
**Upregulated genes**			
1	*il11a*	interleukin 11a	+6.27
2	*CU469544.1-201*	CU469544.1-201	+5.70
3	*junba*	jun B proto-oncogene a	+5.28
4	*fgf20a*	fibroblast growth factor 20a	+4.83
5	*adam8a*	ADAM metallopeptidase domain 8a	+4.82
6	*aldh1a2*	aldehyde dehydrogenase 1 family, member A2	+3.93
7	*irf8*	interferon regulatory factor 8	+3.78
8	*hrh3*	histamine receptor H3	+3.74
9	*grn1*	granulin 1	+3.72
10	*fn1b*	fibronectin 1b	+3.71
11	*stm*	starmaker	+3.63
12	*socs3b*	suppressor of cytokine signaling 3b	+3.59
13	*fstl3*	follistatin-like 3 (secreted glycoprotein)	+3.57
14	*tgm2l*	transglutaminase 2, like	+3.37
15	*LOC565793*	LOC565793	+3.34
16	*pth1a*	parathyroid hormone 1a	+3.19
17	*slc20a1a*	solute carrier family 20, member 1a	+3.04
18	*LOC100006896*	LOC100006896	+3.03
19	*cbx7a*	chromobox homolog 7a	+2.85
20	*c6*	complement component 6	+2.83
21	*serpine1*	serpin peptidase inhibitor, clade E (nexin, plasminogen activator inhibitor type 1), member 1	+2.81
22	*mmp13a*	matrix metallopeptidase 13a	+2.78
23	*vcana*	versican a	+2.75
24	*cygb1*	Cytoglobin-1.	+2.73
25	*cmklr1*	chemokine-like receptor 1	+2.68
26	*rrad*	Ras-related associated with diabetes	+2.66
27	*zgc:158446*	zgc:158446	+2.66
28	*nuak1a*	NUAK family, SNF1-like kinase, 1a	+2.65
29	*fstb*	follistatin b, transcript variant 1	+2.53
30	*fb73b05.x1*	Zebrafish WashU MPIMG EST Danio rerio cDNA clone	+2.52
31	*lepb*	leptin b	+2.46
32	*rerglb*	RERG/RAS-like b	+2.46
33	*f2r*	coagulation factor II (thrombin) receptor	+2.44
34	*bcl2l10*	BCL2-like 10 (apoptosis facilitator)	+2.43
35	*zgc:172120*	zgc:172120	+2.37
36	*ecm2*	extracellular matrix protein 2, female organ and adipocyte specific	+2.36
37	*chtf8*	CTF8, chromosome transmission fidelity factor 8 homolog (S. cerevisiae)	+2.36
38	*alpi.1*	alkaline phosphatase, intestinal, tandem duplicate 1	+2.35
39	*dkk3b*	dickkopf WNT signaling pathway inhibitor 3b	+2.31
40	*htra1b*	HtrA serine peptidase 1b	+2.28
41	*cyth4b*	Cytohesin-4 (PH, SEC7 and coiled-coil domain-containing protein 4)	+2.27

42	*LOC562475*	LOC562475	+2.19
43	*slc16a9a*	solute carrier family 16, member 9a	+2.13
44	*tubb5*	tubulin, beta 5	+2.08
45	*RZ150A3E03.T7*	Zebrafish Kidney Marrow cDNA library Danio rerio cDNA clone RZ150A3E03 3-, mRNA sequence	+2.06
46	*zgc:158334*	hypothetical protein LOC791137	+2.02
47	*mep1a.1*	meprin A, alpha (PABA peptide hydrolase), tandem duplicate 1	+2.02
48	*sfrp1b*	secreted frizzled-related protein 1b	+2.00
49	*LOC796217*	LOC796217	+1.99
50	*ass1*	argininosuccinate synthase 1	+1.98
51	*lamc3*	laminin, gamma 3	+1.97
52	*papss1*	3'-phosphoadenosine 5'-phosphosulfate synthase 1	+1.95
53	*rnpep*	arginyl aminopeptidase (aminopeptidase B)	+1.94
54	*htra3a*	HtrA serine peptidase 3a	+1.94
55	*hpcal4*	hippocalcin like 4	+1.94
56	*entpd4*	ectonucleoside triphosphate diphosphohydrolase 4	+1.94
57	*mvp*	major vault protein	+1.93
58	*furina*	furin (paired basic amino acid cleaving enzyme) a	+1.93
59	*flvcr2b*	feline leukemia virus subgroup C cellular receptor family, member 2b	+1.92
60	*chsy1*	chondroitin sulfate synthase 1	+1.89
61	*rnf145a*	ring finger protein 145a	+1.88
62	*mmp9*	matrix metallopeptidase 9	+1.88
63	*aldh1a3*	aldehyde dehydrogenase 1 family, member A3	+1.86
64	*pdia5*	Protein disulfide-isomerase A5 precursor (EC 5.3.4.1) (Protein disulfide isomerase-related protein)	+1.84
65	*LOC571588*	LOC571588	+1.84
66	*si:ch211-241e1.3*	novel protein laminin subunit alpha-3 (LAMA3)	+1.84
67	*cmpk*	cytidylate kinase	+1.82
68	*LOC100002292*	LOC100002292	+1.80
69	*pltp*	phospholipid transfer protein	+1.80
70	*scg3*	secretogranin III	+1.79
71	*tubb6*	tubulin, beta 6 class V	+1.79
72	*gad45ga*	growth arrest and DNA-damage-inducible, gamma a	+1.79
73	*clu*	clusterin	+1.79
74	*ptprua*	protein tyrosine phosphatase, receptor type, U, a	+1.79
75	*scg3*	Secretogranin-3 precursor (Secretogranin III) (SgIII)	+1.78
76	*si:ch211-137i24.10*	novel protein similar to vertebrate transmembrane 4 L six family member 4 (TM4SF4)	+1.78
77	*fyb*	FYN Binding protein	+1.77
78	*timp2b*	Metalloproteinase inhibitor 2 precursor (TIMP-2) (Tissue inhibitor of metalloproteinases 2) (CSC-21K)	+1.76
79	*ehhadh*	enoyl-CoA, hydratase/3-hydroxyacyl CoA dehydrogenase	+1.73
80	*itga5*	integrin, alpha 5 (fibronectin receptor, alpha polypeptide)	+1.73
81	*creld2*	cysteine-rich with EGF-like domains 2	+1.73
82	*has3*	hyaluronan synthase 3	+1.73
83	*adamts15a*	ADAM metallopeptidase with thrombospondin type 1, motif, 15a	+1.71
84	*cpn1*	carboxypeptidase N, polypeptide 1	+1.70
85	*mamdc2a*	MAM domain containing 2a	+1.67
86	*dpp3*	dipeptidyl-peptidase 3	+1.67
87	*col14a1a*	collagen, type XIV, alpha 1a	+1.65

88	*rasgef1bb*	RasGEF domain family, member 1Bb	+1.65
89	*homeza*	homeobox and leucine zipper encoding a	+1.64
90	*tnni1b*	troponin I type 1b (skeletal, slow)	+1.59
91	*rad52*	RAD52 homolog, DNA repair protein	+1.59
92	*snx18a*	sorting nexin 18a	+1.58
93	*zgc:77147*	zgc:77147	+1.57
94	*lman1*	lectin, mannose-binding, 1	+1.56
95	*slc25a25a*	solute carrier family 25 (mitochondrial carrier; phosphate carrier), member 25a	+1.52
96	*b4galt7*	xylosylprotein beta 1,4-galactosyltransferase, polypeptide 7 (galactosyltransferase I)	+1.52
97	*Q1LYC0_DANRE*	Novel protein (Fragment)	+1.51
**Downregulated genes**			
1	*si:busm1-71b9.3*	si:busm1-71b9.3 (si:busm1-71b9.3), misc RNA	-3.21
2	*si:busm1-71b9*	novel protein similar to zebrafish epithelial cadherin 1 (cdh1)	-3.01
3	*dkk1b*	dickkopf WNT signaling pathway inhibitor 1b	-2.96
4	*aanat2*	arylalkylamine N-acetyltransferase	-2.76
5	*zgc:153154*	zgc:153154	-2.70
6	*astl*	astacin-like metallo-endopeptidase (M12 family)	-2.42
7	*fq27b03.x3*	zebrafish adult brain cDNA clone IMAGE:4832573 3-, mRNA sequence	-2.40
8	*prf1.5*	perforin 1.5	-2.39
9	*fx06c11.x1*	Gong zebrafish ovary cDNA clone IMAGE:5619069 3-, mRNA sequence	-2.26
10	*si:ch211-238g23.1*	novel protein kinase domain containing protein	-2.23
11	*LOC100004613*	LOC100004613	-2.22
12	*aqp10a*	aquaporin 10a	-2.20
13	*fv44e07.x1*	Sugano SJD adult male cDNA clone IMAGE:5411413 3- similar to SW:PEX_MOUSE P70669 METALLOENDOPEPTIDASE HOMOLOG PEX, mRNA sequence	-2.16
14	*si:dkeyp-79b7.4*	novel protein	-2.15
15	*ucp1*	uncoupling protein 1	-2.07
16	*zgc:110712*	novel keratin protein (zgc:110712)	-2.04
17	*atp8b3*	ATPase, aminophospholipid transporter, class I, type 8B, member 3	-2.03
18	*fjx1*	four jointed box 1	-2.02
19	*slc10a1*	solute carrier family 10 (sodium/bile acid cotransporter), member 1	-1.97
20	*lamb2l*	laminin, beta 2-like	-1.94
21	*zgc:113307*	zgc:113307	-1.94
22	*LOC556634*	LOC556634	-1.93
23	*LOC100001952*	LOC100001952	-1.87
24	*smim8*	small integral membrane protein 8	-1.87
25	*gyg1b*	glycogenin 1b	-1.84
26	*anxa1c*	annexin A1c	-1.83
27	*egfl7*	EGF-like-domain, multiple 7	-1.83
28	*tm7sf2*	transmembrane 7 superfamily member 2	-1.83
29	*Q1MT48_DANRE*	Novel protein similar to vertebrate transducin-like enhancer family (E(Sp1) homolog Drosophila) (Fragment)	-1.79
30	*si:dkey-73p2.2*	si:dkey-73p2.2	-1.78
31	*eif43bp3*	eukaryotic translation initiation factor 4E binding protein 3	-1.75
32	*LOC560375*	LOC560375	-1.72

33	*scn4ba*	sodium channel, voltage-gated, type IV, beta a	-1.70
34	*tuba7l*	tubulin, alpha 7 like	-1.70
35	*p2rx1*	purinergic receptor P2X, ligand-gated ion channel, 1	-1.64
36	*ankrd22*	ankyrin repeat domain 22	-1.64
37	*LOC100003522*	LOC100003522	-1.61
38	*si:dkeyp-32b1.1*	novel protein similar to vertebrate lysyl oxidase-like 2 (LOXL2)	-1.61
39	*si:dkey-98p3.2*	si:dkey-98p3.2	-1.61
40	*phex*	phosphate regulating endopeptidase homolog, X-linked	-1.60
41	*dpt*	dermatopontin	-1.59
42	*ugt5a5*	UDP glucuronosyltransferase 5 family, polypeptide A5	-1.58
43	*slc43a1b*	solute carrier family 43 (amino acid system L transporter), member 1b	-1.58
44	*zgc:92360*	hypothetical protein LOC436988	-1.52
45	*tmtopsb*	teleost multiple tissue opsin b	-1.50
